# Safety and Innovation in Conventional Plastics: A Review of Polymer Synthesis and Emerging Technologies

**DOI:** 10.3390/polym18081007

**Published:** 2026-04-21

**Authors:** Derval dos Santos Rosa, Hélio Wiebeck, Alana Gabrieli Souza, Sueli Aparecida de Oliveira, Manoel Lisboa da Silva Neto

**Affiliations:** 1 Center for Engineering, Modeling, and Applied Social Sciences (CECS), Federal University of ABC (UFABC), Santo Andre 09280-560, Brazil; derval.rosa@ufabc.edu.br (D.d.S.R.); suoliv@uol.com.br (S.A.d.O.); 2 Metallurgical and Materials Engineering Department (PMT), Polytechnic School, University of Sao Paulo (USP), Sao Paulo 05508-010, Brazil; hwiebeck@usp.br (H.W.); manoel.lisboa@mlsn.com.br (M.L.d.S.N.)

**Keywords:** BPA-free polymers, polymer synthesis, ecological risk, bio-based feedstocks and routes, environmental impact

## Abstract

Persistent misconceptions about the alleged presence of bisphenol A (BPA) in major commodity plastics continue to distort public perception and, in some cases, regulatory discourse. This occurs despite scientific evidence showing that these polymers are synthesized without BPA. This review examines five widely used plastics—PET, PE, PP, PS, and PVC—focusing on their synthesis, structure–property relationships, and technological changes affecting the sector. We highlight recent innovations in green catalysis, bio-based feedstocks, polymer redesign, and advanced recycling. These advances are speeding the shift to efficient, sustainable processes and a circular polymer economy. We discuss market trends and regulatory frameworks to explain their global and Brazilian relevance, showing how communication gaps can lead to misinformation. By uniting chemical, technological, and regulatory views, this review supports public understanding, evidence-based policy, and the development of safer, high-performance, sustainable polymers.

## 1. Introduction

Synthetic polymers are among the most strategically important classes of materials in contemporary society. Their low density, tunable properties, chemical stability, and compatibility with large-scale continuous manufacturing have enabled their integration into essential sectors, such as food packaging, transportation, energy, construction, healthcare, and consumer goods [1]. Among these materials, polyethylene terephthalate (PET), polyethylene (PE), polypropylene (PP), polystyrene (PS), and polyvinyl chloride (PVC) dominate global production and represent the core of the plastics value chain [2]. The selection of these polymers in this review is based on their dominant contribution to global plastic production, their extensive use across critical industrial sectors, and the maturity of their industrial synthesis routes [3]. According to the European Plastic Production (2024), polymer production was as follows: PE 22%, PP 16%, PVC 9%, PS 5%, and PET 5% [4]. Together, they account for the vast majority of commodity plastics worldwide and encompass the main paradigms of polymerization, including step-growth polymerization, free-radical processes, and coordination catalysis. Their widespread presence in applications with direct human and environmental exposure places them at the center of regulatory frameworks and public perception debates, making them highly representative systems for evaluating both technological developments and safety considerations [5].

Their ubiquity has increased public scrutiny of chemical safety and environmental sustainability. This scrutiny poses complex questions in polymer chemistry, toxicology, regulation, and industrial innovation. Concern about bisphenol A (BPA) in plastic products is growing, fueled by its status as an endocrine disruptor and its possible effects on human health. BPA is a precursor in the production of polycarbonate materials and epoxy systems. However, the belief that all plastics contain BPA is a persistent misconception, repeated in the media, in regulations, and in consumer debates. This generalization remains despite extensive evidence that the polymers discussed above use catalytic or radical mechanisms and do not involve BPA or create bisphenolic intermediates.

At the same time, the polymer industry is undergoing an unprecedented shift driven by sustainability policies, decarbonization targets, market pressures, and advancements in catalysis and process intensification. These technological developments are reshaping polymer production and end-of-life management through innovations in reaction engineering, advanced monomer purification, renewable feedstock routes, additive substitutes with improved toxicological profiles, and chemical and enzymatic recycling technologies that regenerate high-purity monomers. Together, these advances not only influence performance and circularity but also reinforce the safety and traceability of conventional polymers, helping consolidate evidence-based frameworks for assessing human exposure and environmental impact.

Recent regulatory changes in the European Union, the United States, and Brazil have placed increased emphasis on chemical safety, transparency, and traceability as core requirements for polymer production and use. For example, the European Union’s Regulation (EU) No 10/2011 governs plastic materials intended to come into contact with food, while the United States Food and Drug Administration (FDA) enforces standards under Title 21 of the Code of Federal Regulations. Brazil regulates polymers through ANVISA Resolution RDC No. 56/2012 for food-contact materials. These major agencies and frameworks have updated standards on food-contact materials, additive restrictions, analytical testing, and certification. Current regulatory frameworks require stricter evaluation of substances with migration potential, demand improved detection of non-intentionally added substances, and seek alignment with circular-economy goals [6]. Producers are now required to integrate compliance strategies with innovation to enhance the adoption of cleaner synthesis, advanced stabilization, and improved recycling processes.

Despite these advances, critical disconnection persists. Regulatory bodies and the peer-reviewed literature confirm that major commodity polymers do not contain BPA in their molecular structure or synthetic routes. Yet public perception often remains anchored in outdated or misleading information. This discrepancy affects consumer trust, policy debates, and industrial decision-making. A rigorous and accessible synthesis of scientific, technological, and regulatory evidence is needed to reestablish factual consistency across sectors. However, the absence of BPA in polymer synthesis does not guarantee overall material safety [7]. The final safety of plastics is strongly influenced by formulation components, including additives such as plasticizers, stabilizers, antioxidants, and processing aids, as well as non-intentionally added substances from manufacturing, degradation, or recycling processes [8,9].

This review integrates the scientific literature, patents, market analysis, and regulations to explain the absence of BPA in the synthesis of major commodity polymers, using precise chemical methods. It aims to address the literature gap where technical chemical evidence and public perception often do not align. The review describes conventional and emerging production technologies, catalyst systems, and structure–property relationships. It connects these topics to global and Brazilian markets and evolving safety and sustainability regulations. We highlight how catalysis, renewable monomers, polymer redesign, and advanced recycling change the plastics industry. This integrated perspective offers an evidence-based view to dispel misconceptions, guide regulatory and industrial decisions, and highlight strategic priorities—such as safety-by-design, circularity, and effective scientific communication. Along with summarizing recent advances, the review evaluates the limitations, trade-offs, and industrial feasibility of new technologies, aiming to bridge the gap between lab-scale innovation and large-scale deployment.

## 2. Synthesis of Conventional Polymers

Before examining the synthesis routes of common polymers, it is important to explain the relevance of this survey. Conventional plastics, such as PET, HDPE, LDPE, PP, PS, and PVC, are used mainly in packaging, household appliances, and industrial devices. The following subsections will outline how each polymer is obtained, highlighting reagents, catalysts, and operating conditions. For PET, HDPE, LDPE, PP, PS, and PVC, differences between condensation polymerization, radical addition, and Ziegler–Natta/metallocene catalysts will be addressed, along with recent advances in catalysis and energy efficiency. Research into high-performance catalysts has also grown significantly in recent years.

### 2.1. Polyethylene Terephthalate (PET)

Polyethylene terephthalate, CAS number 25038-59-9, is a widely produced thermoplastic, used in textile fibers, electronics, packaging, fabrics, and other materials [10,11], with chemical inertness, light weight, and transparency. PET is especially relevant because, although its importance is recognized, PET is frequently and erroneously associated with bisphenol A (BPA). Regulatory agencies (FDA, EFSA, and NIEHS) and the scientific literature consistently confirm that BPA is not used in PET synthesis and is not generated as a degradation product during conventional processing or consumer use. PET’s molecular structure does not contain phenolic monomers or BPA analogs, and properly manufactured PET complies with global food-contact regulations without requiring BPA-free reformulations. Nevertheless, safety assessments of PET materials must also consider additives, degradation products, and potential contaminants introduced during processing or recycling, which may influence migration behavior and overall material safety [6,12].

**Figure 1:**
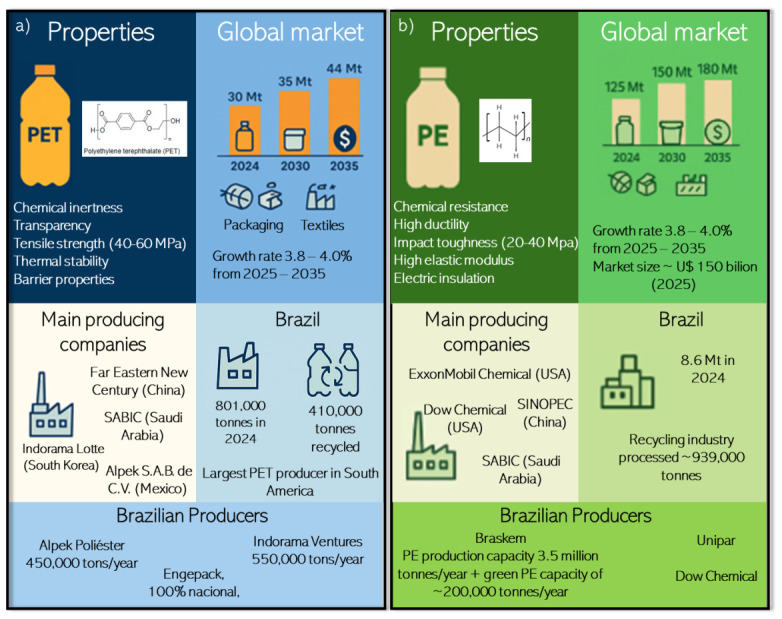
(**a**) Polyethylene terephthalate (PET) and (**b**) polyethylene (PE) overview: properties, global market growth (30 Mt in 2024 to 44 Mt in 2035), main producing companies, and Brazil’s main numbers.

As summarized in Figure 1, the global PET market size was 30 Mt in 2024, and is expected to grow to 44 Mt by 2035, with a compound annual growth rate (CAGR) of 3.8% from 2025 to 2035 [13,14]. Specific segments, such as PET bottles, continue to grow and account for a large share of the volumetric market. Considering market value, the global PET market was estimated at ~US$ 39–42 billion by mid-2025 and is projected to reach ~US$ 68–70 billion by mid-2030–2035, driven by food and beverage packaging, films, and textile applications [15]. The leading producing companies are Indorama Lotte Chemical Corporation (South Korea), Ventures Public Company Limited (Thailand), Reliance Industries Limited (India), Far Eastern New Century Corporation (China), SABIC (Saudi Arabia), Jiangsu Sanfangxiang Group (China), and Alpek S.A.B. de C.V. (Mexico) [15,16]. The Supplementary Material also described Brazil’s relevance in the PET market and the large numbers associated with production capacity and recycling.

#### 2.1.1. PET Common Synthesis Routes

PET can be synthesized through three well-established industrial routes by combining terephthalic acid and its derivatives with ethylene glycol to form long polyester chains. The differences lie in the starting monomer form, reaction sequence, conditions under which PET is produced, and the technologies used to produce PET. The technologies used in PET production are presented in the Supplementary Material (Table S1), along with detailed descriptions of the common routes.

**Figure 2:**
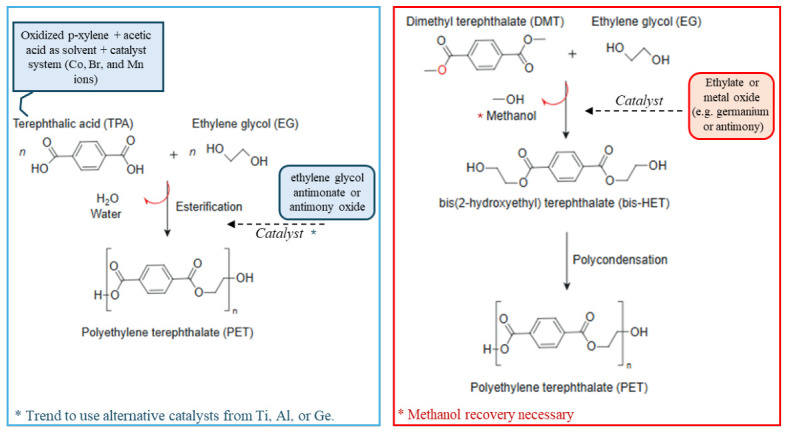
An illustration of the two most common synthesis routes for PET obtention.

Esterification is the most common continuous synthesis route, employing purified (dimethyl)terephthalic acid (TPA), ethylene glycol (EG), and a catalyst. The catalyst and process conditions can significantly influence the polymer’s properties and efficiency, with a trend toward the use of more sustainable alternatives [17]. This process is highly productive and considered to have lower raw material costs. However, it requires efficient removal of byproducts to avoid degradation. Transesterification (DMT Route) + polycondensation is less commonly used due to the need for methanol recovery and high production costs. It was the dominant route in the 1970s–1980s, but lost ground to the TPA route due to economic and environmental issues. In this route, dimethyl terephthalate (DMT) reacts with EG using ethylate or a metal oxide catalyst at 150–200 °C. After polycondensation, PET and ethylene glycol are generated through transesterification. The two common synthesis routes are presented in Figure 2.

In the PET manufacturing context, the integrated PTA-PET route combines two routes in the same production site: i. p-xylene oxidation and terephthalic acid purification, followed by ii. PTA + MEG conversion into PET (direct esterification/pre-polycondensation, followed by melt polycondensation) [18]. The key advantages of method 3 are logistics and quality control, as it eliminates intermediate PTA transport and storage, lowers handling costs, and mitigates moisture/trace-metal pickup that can degrade intrinsic viscosity, color, and acetaldehyde levels [19]. The main trade-offs are higher upfront CAPEX, added operational complexity across units, and reduced flexibility to buy PTA on the spot market. Overall, at world-scale capacities, the integrated PTA-PET complex typically delivers lower unit OPEX and contamination risk than direct esterification with imported PTA, while retaining the processing steps and by-product profile listed for the direct esterification route [20].

#### 2.1.2. PET Alternative Synthesis Routes

Several alternatives have been investigated over the past few decades to address environmental, economic, and performance-related challenges in PET production. These approaches often aim to reduce reliance on fossil-derived raw materials, improve process efficiency, or enhance recyclability. The most studied strategies are bio-based pathways for producing monomers from renewable feedstocks (Figure S2), catalytic innovations to reduce energy consumption and by-product formation, and chemical recycling processes. Sandei et al. (2022) reported that bio-based EG production is the main obstacle, as it is derived from bioethanol and competes with food products, thereby affecting the final price [21]. Kim et al. (2019) investigated the effect of a Ti–catalyst in polycondensation for PET fiber development [22]. Ti-PET showed a molecular weight similar to that of conventional PET, with lower crystallinity and tensile strength, indicating that the catalysts influence the fibers’ thermomechanical properties. Wang et al. (2022) researched an aluminum-based catalyst for PET synthesis and reported an optimal content of 0.05% with a reaction time of 150 min at 280 °C [23].

Marshall et al. (2022) studied the use of 2,5-furandicarboxylic acid as a TPA substitute for PET synthesis, yielding poly(ethylene furanoate) (FDCA), a bio-based polyester with superior barrier and thermal properties [24]. The strategic relevance of FDCA is underscored by its early identification by the U.S. Department of Energy as a priority biomass-derived platform molecule with strong potential for polyester applications [25]. Xu et al. (2017) prepared ethylene glycol from cellulose using a tungsten-based catalyst, yielding 58%, providing a new pathway to mitigate dependence on fossil resources [26]. Kwon and Lee (2024) prepared a review describing carbon-neutral routes for PET synthesis, highlighting the emergence of promising catalytic routes [16]. Berger and Pfeifer (2025) [27] prepared bio-PET from wheat straw ethanol and corn stover isobutene. They evaluated costs and carbon neutrality and reported a decrease in carbon footprint from 2.99 to 0.46 kg CO_2_ eq./kg for bio-PET fibers.

Research trends include the production of aromatic monomers from biomass, such as bio-p-xylene synthesis from platform molecules like 5-hydroxymethylfurfural (HMF), isoprenoids, and lignin derivatives. Despite significant progress, issues such as catalyst deactivation, coke deposition, and the high energy demand of deoxygenation steps remain major challenges. Design principles based on hierarchical catalyst structures and regeneration strategies are proving effective in mitigating coke formation and improving long-term catalyst stability [28]. Additionally, carbon-neutral PET and structural redesign of polyesters to enable more efficient closed-loop recycling are widely under investigation. Catalytic conversion strategies for bio-derived TPA and EG show promising results, although techno-economic viability and catalyst lifetimes still constrain large-scale implementation [16]. Beyond feedstock substitution, polymer redesign itself offers a way to circularity: terephthalic polyesters incorporating longer methylene diols exhibit improved ductility and can be chemically depolymerized under milder methanolysis conditions (e.g., 140 °C), enabling efficient monomer recovery for closed-loop reuse [29]. Figure S1 illustrates alternative PET synthesis routes, highlighting the renewable origins of ethylene glycol and terephthalic acid. EG can be produced from glucose via glycolaldehyde, or from ethanol and ethylene derived from biomass and CO_2_, highlighting the potential of carbohydrate-based feedstocks to replace fossil-derived ethylene.

The literature reports direct conversion from lignocellulose (e.g., corn stalks, miscanthus, and poplar wood) using tungsten catalyst in two steps: hydrolysis and cellulose hydrogenation to glucose formation [30]. In this route, cellulose hydrolysis yields oligosaccharides and glucose, which are then catalytically degraded by a retroaldol reaction into glycolaldehyde (GA). Then, EG is produced by GA hydrogenation, and fructose yields propylene glycol and glycerol [26]. Although this route is environmentally interesting, the yield is low, ranging from 10 to 20%. TPA can be synthesized from p-xylene, which itself may originate from diverse bio-based intermediates, such as isobutanol, propane, furane derivatives, or benzene, all ultimately traceable to glucose as a carbon source. Anellotech, in collaboration with its development partners IFPEN and Axens, developed a technology to produce p-xylene from biomass pyrolysis, called “Biomass to AromaticsTM”. The process comprises three steps, and the yield ranges from 30 to 47% [31]. Bioforming^®^ technology, protected by several patents, was developed by Virent [32], which also results in the obtention of p-xylene from biomass. In 2011, a turning point occurred following a collaboration between Virent and Coca-Cola to develop 100% bio-based PET bottles, named PlantBottle [33].

Additionally, routes involving p-cymene (PCY) and terpenes, such as limonene extracted from citrus residues, have gained attention as promising renewable pathways. SABIC Innovative Plastics patented PCY synthesis from terpenes and its conversion into TPA [34]. Its advantages lie in the use of renewable, widely available agro-industrial residues, such as citrus peels, reducing dependence on fossil-derived aromatics and contributing to waste valorization. Moreover, the transformation of limonene into p-cymene and subsequently into terephthalic acid can be integrated with existing industrial platforms for fragrances and fine chemicals. From an environmental perspective, this pathway aligns with green chemistry principles by lowering carbon footprint and energy intensity compared with conventional petrochemical processes [27]. Importantly, bio-based PET obtained via the SABIC process is free from bisphenol A (BPA), reinforcing its suitability for safe food and beverage packaging applications. Figure S2 illustrates the proposed SABIC reactions and yields (~84%) [21]. Other alternatives include succinic and muconic acids, which can also serve as precursors to aromatic monomers. The succinic acid route includes microbial synthesis of glucose, which is at the laboratory stage, as well as malic acid, using *Aspergillus* as the microbe and yielding 69% [35].

Overall, although these alternative routes demonstrate significant potential to reduce fossil dependence and enable renewable integration, their large-scale implementation remains constrained by catalyst stability, feedstock availability, and process economics [36]. Compared to conventional petrochemical routes, these alternatives are still limited in scalability, highlighting a critical gap between laboratory innovation and industrial deployment [37].

#### 2.1.3. Recent Breakthroughs (2023–2025)

Recent progress in PET recycling and upcycling has accelerated toward higher selectivity, lower energy consumption, and expanded product value. The findings below represent step changes in both mechanistic understanding and practical implementation.

- Oxygen-vacancy-driven selective oxidation (2024): Au/NiO catalysts enabled PET depolymerization into TPA (99% yield), and glycolic acid (GA, 88% yield), showing that catalyst electronic/defect engineering can tune product distribution and promote upcycling toward oxygenates rather than only monomers [38]. In this case, oxygen vacancies in NiO facilitate the activation of O_2_ and the transfer of lattice oxygen to PET cleavage sites, while Au sites promote the selective oxidation of glycol fragments to GA and stabilize the intermediates.
- Thermochemical—electrochemical cascade (2024): PET conversion into glycine (~75% yield) without H_2_, through oxidative depolymerization to oxalic acid followed by electroreduction C-C/C-N bond-forming steps on TiO_2_ electrodes. This method demonstrates a paradigm for combining thermal catalysis and electrochemistry to make N-containing fine chemicals from waste plastics [39].
- Polymer design for mild closed-loop recycling (2024): medium and long-chain terephthalic polyesters that depolymerize at mild temperatures (~140 °C in methanol) embody a materials-by-design approach in which polymer structures are intentionally engineered at the molecular level by monomer selection, functional group incorporation, or chain architecture, enabling targeted properties, including improved recyclability, depolymerization under mild conditions, and enhanced performance [29].
- Value-added material upcycling (2024): conversion of discarded PET into flexible, corrosion-resistant materials, providing circularity pathways where monomer recovery is not feasible [40].

Several mechanistic levers can be identified as recurring drivers of improved PET valorization. These include ester groups, Lewis-acid activation, oxygen-vacancy-mediated oxidation, coupled thermochemical–electrochemical cascades for redox-intensive transformations, and enzyme catalysis specifically tailored to address mixed plastic streams [41]. These strategies highlight how catalyst site design, defect engineering, and hybrid catalytic platforms can redirect depolymerization pathways and expand product selectivity beyond conventional monomers [38,39,42,43]. Techno-economic and life-cycle analyses, particularly in cascade catalysis studies, indicate that such hybrid routes hold potential to be both cost-effective and low-carbon at scale [39]. Complementing these findings, recent reviews emphasize the central role of heterogeneous solid catalysts in lowering depolymerization severity and enabling diverse routes, such as alcoholysis, glycolysis, pyrolysis, and reductive conversions, under milder conditions, where the structure–activity relationship of catalyst active sites emerges as a critical design lever for selectivity and process compatibility [42]. Despite these advances, most of these strategies remain at early development stages, with limited demonstration under industrially relevant conditions. Challenges related to catalyst durability, process integration, and economic feasibility still hinder large-scale adoption.

Karkanis et al. (2025) [44] used PET waste to prepare dental resins and conducted liquid chromatography to guarantee the safety of the developed material, considering human health and environmental friendliness. The authors reported that their PET resin did not release bisphenol A or other contaminants and demonstrated significant hardening and mechanical performance [44]. Bittner et al. (2014) investigated the human safety of several commodity plastics and reported that PET is one of the safest thermoplastic resins, with no detectable hazardous chemicals released [45].

Despite the significant advances in PET depolymerization and upcycling technologies, important limitations still constrain their large-scale implementation. One of the main challenges is the need for well-sorted, high-purity feedstocks, as the presence of other polymers, additives, colorants, and contaminants can interfere with catalytic activity, reduce reaction selectivity, and compromise the quality of the recovered monomers [46]. For this reason, several technologies need extraction or dissolution as pretreatment, followed by PET dissolution [47]. The limitations of PET chemical recycling are reflected in the low EU chemical recycling capacity, reported at 3.6% in 2022, based on the total rate of recycled PET produced [48]. McNeeley et al. (2025) reported limitations in costs, environmental impact, and resource consumption, highlighting a research gap in technologies to maximize textile feedstock processes due to the presence of different dye colors [46]. Guo, Wu, and Wang (2025) suggested that future efforts should focus on developing greener solvents or catalysts to reduce costs and waste and enhance process yield [49].

### 2.2. High- and Low-Density Polyethylene (HDPE and LDPE)

Polyethylene is one of the most widely produced synthetic polymers worldwide (CAS 9002-88-4), with annual production exceeding 100 million tons [50]. Structurally, PE is composed of long ethylene chains, and depending on the degree of branching and molecular weight distribution, it is classified into low-density polyethylene (LDPE), linear low-density polyethylene (LLDPE), and high-density polyethylene (HDPE) [51,52]. This polymer has excellent chemical resistance, high ductility, impact toughness (20–40 MPa), an elastic modulus (200–1500 MPa), and a processing temperature range of −80 to 120 °C [52,53].

The global PE market reached approximately 120–125 Mt in 2024 and is projected to reach 150 Mt by 2030, reflecting a CAGR of ~3.5–4.0% [13,54]. Additionally, the global PE market is estimated at ~US$ 140 billion in 2025 and is expected to surpass US$ 200 billion by 2035, driven primarily by packaging, infrastructure, and emerging applications in renewable energy and medical devices [55,56]. The leading PE producers include ExxonMobil Chemical (USA) [57], LyondellBasell (Netherlands) [58], Sinopec (China) [59], Saudi Basic Industries Corporation—SABIC (Saudi Arabia) [60], and Dow Chemical (USA) [61], among others. The Supplementary Material also described Brazil’s relevance in the PE and bio-PE markets and the large numbers associated with production capacity and recycling.

#### 2.2.1. PE Common Synthesis Routes

**Figure 3:**
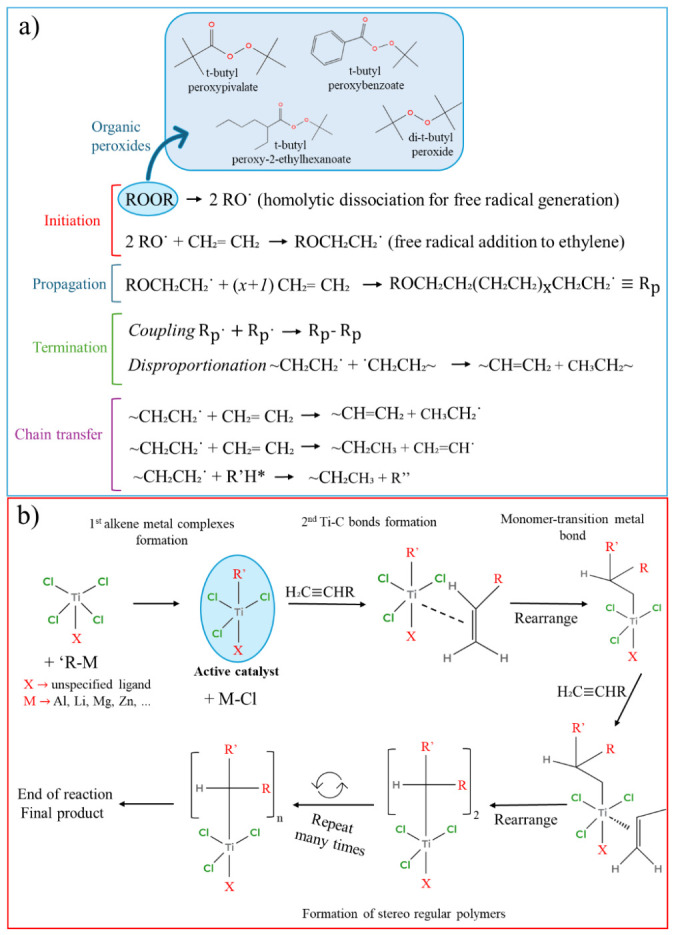
Schematic overview of polyethylene synthesis. (**a**) High-pressure free-radical route, and (**b**) Ziegler–Natta coordination–insertion cycle. Note: the * presented with hydrogen represents the excited state of the molecule.

Historically, PE was the first synthetic hydrocarbon to be polymerized via free-radical polymerization under high pressure; however, following several explosions, other methods were investigated at lower pressure and with different catalysts [62]. Ziegler–Natta (ZN) synthesis is the most common method, carried out under medium pressure and organic catalytic conditions, while Phillips’ process uses chromium oxide–silica gel as a catalyst. Detailed descriptions of the common routes are also provided in the Supplementary Material. Briefly, PE is synthesized from ethylene (CH_2_=CH_2_) via two distinct routes: free-radical polymerization at very high pressure for LDPE, and coordination catalysis at moderate pressure for LLDPE and HDPE. Figure 3a shows the free-radical polymerization route and its main steps of initiation, propagation, termination, and chain transfer, with an explanation in the Supplementary Material.

The second industrial technologies family involves transition-metal catalysts to coordinate and insert ethylene into a metal–carbon bond, enabling precise control over chain transfer, comonomer incorporation, and molecular-weight distribution. Figure 3b illustrates the ZN route and the main steps involved in PE synthesis, including the formation of the active catalyst with M-Cl, followed by chemical reactions that result in polymer chain segments [-CH_2_-CH(R)-]n bound to titanium.

Two other industrial processes can be cited: Phillips (Cr/SiO_2_) and single-site catalysts [63]. Phillips catalysts are based on chromium species dispersed on a refractory oxide, typically generated by impregnation followed by thermal activation [64].

Thermal activation generates surface chromate/chromyl sites that, under polymerization conditions, evolve into Cr–alkyl active centers capable of ethylene Cossee–Arlman coordination–insertion without cocatalyst [65], resulting in operational simplicity, impurity tolerance, and broad molecular weight distribution [63]. Catalyst productivity and molecular weight are tuned by activation protocol, hydrogen dosage, and reactor residence time. The technology’s robustness enables stable operation with standard cracker-grade ethylene and modest comonomer levels during LLDPE production [66].

Single-site catalysts (SSCs) comprise well-defined metal centers, usually metallocenes (-bonded organometallics) as Cp_2_ZrCl_2_ and, increasingly, post-metallocenes, as pyridine–imine or α-diimine Fe/Ni, that polymerize ethylene via a single, uniform type of active site. After activation, the metal–alkyl center inserts ethylene and α-olefin comonomers, yielding narrow molecular weight and uniform short-chain branching [67], resulting in precise microstructure [63]. However, some limitations include high activator cost and sensitivity to impurities. The synthesis routes reviewed in this section are presented in Table S2, along with the main characteristics of each route and its processing characteristics.

#### 2.2.2. PE Alternative Synthesis Routes

Beyond the well-known methods, several alternative feedstock pathways can yield ethylene as a drop-in monomer for standard PE platforms (ZN/Phillips/single-site). The bio-ethylene from ethanol dehydration is the most common method. In this method, lignocellulosic materials, such as wood, corn, or sugarcane bagasse, are fractionated and purified, and cellulose is converted to glucose via enzymatic hydrolysis. Then, the sugars are fermented to produce bioethanol, which is dehydrated over Al_2_O_3_/zeolitic catalysts (300–450 °C) to produce bio-PE [68]. Polymerization occurs through -bonds in the ethylene monomer, which break to form new bonds with similar monomers, resulting in a polymeric chain, with hydrogen controlling the molecular weight. Typical operational challenges include catalysts, water management, and regional constraints related to land use and feedstock costs.

Another environmentally friendly method is the use of biogas. Three routes are known to convert methane into ethylene. The first one converts CH_4_ to synthesis gas (syngas), a mixture of CO and H_2_ via partial oxidation of biomass, transformed into methanol (using catalysts such as Cu, ZnO, or Al_2_O_3_) and, subsequently, olefins, such as C_2_H_4_ or C_3_H_6_, to generate ethylene [69], with a yield between 35 and 45%. The second route is similar, but olefin formation does not occur, and syngas is used to synthesize synthetic naphtha, which is then steam-cracked in a pyrolytic oven (800–850 °C) for milliseconds to generate ethylene, propylene, and other co-products [70,71]. The third route is the OCM (oxidative coupling of methane), which avoids syngas formation and results in ethylene formation via an oxidative reaction with alkali or metallic catalysts [72,73]. The first two routes are at the demo/early commercial stage, whereas OCM remains at pilot scale due to selectivity limitations. These options valorize waste methane and offer low-carbon potential but require CAPEX/energy-intensity hurdles, complex integration, and strict impurity control. Electrification is advancing toward CO_2_-to-ethylene electroreduction, in which Cu-based cathodes convert CO_2_ and water to C_2_H_4_ using renewable power [74,75]. All studies involving electrification highlight lower carbon emissions (~50%). This approach is lab- and pilot-scale yet attractive because it is fully electrifiable and modular. Additionally, PE can be synthesized through alternative industrial pathways that are incorporated into conventional processes. Taken together, these routes demonstrate clear potential for decarbonizing ethylene production. However, most remain constrained by high energy demand, complex process integration, and feedstock variability. Compared to conventional steam cracking, these pathways are not yet competitive at a large scale, underscoring the need for further optimization of catalyst design and system integration.

Considering the catalyst families, the ZN system evolves over time. While the first generation was low in productivity, the evolution involves internal/external donors, such as diethers and silanes, resulting in better control of productivity, hydrogen sensitivity, and important physico-chemical PE properties during polymerization [76]. Most internal donors from industry involve 1,3-diethers (LyondellBasell) [77], 1,3-diol diesters- SABIC (SABIC, LyondellBasell, and Sinopec Corp), alkoxyalkyl esters and alkoxyalkyl 2-propenoate (DOW) [78], 1,8-naphthyl diaroylate (BASF), o-acyloxy phenylacetic acid esters (BASF) [79], and citraconates (Borealis), among others [80].

Phillips catalysts can vary depending on silica pretreatment, calcination temperature, Ti/F modification, and preservation of impurity tolerance. Jongkind et al. (2020) described various metal–alkyl cocatalysts and reported that Cr redox chemistry can be tailored, thereby altering polymer chemistry, induction periods, and catalyst activity [65]. Single-site catalysts create uniform active sites that deliver narrow molecular weight and uniform short-chain branching when copolymerizing with C4–C8 α-olefins, enabling differentiation of LLDPE/VLDPE microstructures [81].

#### 2.2.3. Recent Breakthroughs (2023–2025)

During 2023–2025, several advances reshaped the PE synthesis. Progress concentrates on (i) late-transition-metal catalysis that unlocks new microstructures, (ii) earth-abundant metals transitioning toward industrial scale, (iii) designed long-chain branching through single-catalyst architectures, and (iv) platform innovations (screening and reactor strategies) that shorten discovery-to-plant timelines and broaden asset flexibility.

- Ultra-high molecular weight polyethylenes (UHMWPEs) with molecular weights above 106 g·mol^−1^ exhibit outstanding properties, including high impact resistance and strength, good corrosion resistance, and biocompatibility [82]. For this PE type, catalyst efficiency is essential to achieve adequate properties [83], since early-metal systems exhibit a narrow MWD but struggle with polar comonomers and some UHMW regimes, whereas late-metal platforms fill this gap [84].
- Fe-based catalysts (α-diimine and related ligand frameworks) continue to move from homogeneous proofs-of-concept toward supported/heterogeneous forms compatible with mainstream reactors, with scale-up reports and commercial deployments targeting highly linear PE [85].
- The programmed long-chain branching (LCB) approach via ladder/dual-chain growth is interesting because, in emerging coordination pathways, a single metal center promotes the simultaneous growth of two chains, which are then coupled via a diene, producing controlled LCB in a single catalytic cycle [81]. Using this approach, bubble stability and melt strength are achieved without relying on high pressure. Recent advances demonstrate catalytic pathways that build branched architectures on demand, either in a single metal or by engineering LCB in solution with finely controlled kinetics [86].
- Automated platforms with parallel microreactors, preparation robotics, and in-line analytics accelerate the optimization of ligands, activators, and support protocols, as well as the targeted tuning of α-olefin/oligomer distributions for comonomer slates [87]. This shortens the discovery-to-pilot cycle, improves reproducibility, and enables finer microstructural control during polymerization, accelerating the formation of mono/bi/trimodal architectures and special PEs with predictable properties [88].

These PE synthesis advances widen the industrial design space while leveraging platforms already at world scale. Critically, all these routes polymerize ethylene exclusively, enabling BPA-free materials suitable for food contact when formulated to regulatory specifications [89]. Although PE is a widely studied commodity, there remains a high volume of publications on technological advances, new scientific discoveries, and the search for outstanding properties. Arndt et al. (2024) provided new insights into different reactors and applications, long- and short-chain variations, and branching variations [90]. Their study demonstrated differences in molar mass distribution and properties. While these advances significantly expand the PE design space, their industrial implementation often depends on compatibility with existing infrastructure and economic viability. As a result, conventional catalytic systems remain dominant, highlighting the gap between emerging catalyst innovation and large-scale deployment.

### 2.3. Polypropylene (PP)

Polypropylene (PP, CAS 9003-07-0) emerged in 1958, synthesized via stereospecific coordination catalysis to control tacticity [91]. Its applications include fibers and filaments, home appliances, packaging, medical and electrical devices, automotive parts, interior panels, and more [92]. According to Schwab et al. (2024), annual PP production exceeds 100 million tons [50]. According to Grand View Research, the market size was estimated at USD 123.5 billion in 2022, growing at a CAGR of ~5% from 2023 to 2030 [93]. Asia Pacific is the market leader and, in 2024, accounted for the largest revenue share of 56%. The global PP market companies include LyondellBasell Industries Holdings B.V., BASF SE, ExxonMobil Corporation, China Petroleum & Chemical Corporation, SABIC, Eastman Chemical Company, Trinseo S.A., LG Chem, Total S.A., and Westlake Chemical Corporation [94]. Considering the latest announcements from some companies, in September 2024, Braskem launched bio-circular PP to support the Quick Service Restaurant industry, and in November 2024, Highland Plastics launched Kelvinite 2100, a halogen-free, flame-retardant polypropylene to meet automotive demands. The Supplementary Material also discusses the relevance of Brazil in the PP, and Figure S3 summarizes the key information presented in this section.

#### 2.3.1. PP Common Synthesis Routes

Similar to PE, PP is produced by coordination–insertion polymerization under moderate temperature/pressure using heterogeneous ZN and single-site catalysts [95]. Historically, PP synthesis using the ZN method followed the PE success in 1954, with the preparation of crystalline PP and the determination of its crystal structure. Whereas PE relies on both free-radical and coordination platforms (ZN/Phillips/single-site), PP is almost exclusively made via coordination routes because tacticity (iso/syndio/atactic) must be controlled during propagation [96]. Figure 3b can also be used to describe the PP common synthesis route, once both polyolefins have a similar ZN catalyst mechanism. Additionally, the catalyst architecture and reactor configuration determine comonomer incorporation, chain transfer, and MWD [90]. Detailed descriptions of the common routes are also provided in the Supplementary Material.

Ziegler–Natta PP polymerization is divided into generations based on the catalyst’s generation, as reflected in isotactic content and PP yield. While the first gen showed a ~20 g PP/g catalyst, the third showed a ~50 kg PP/g catalyst, and the same trend held for subsequent generations. Metallocenes and other single-site catalysts were also an option for PP synthesis, developed to complement ZN catalysts, focus on the development of new microstructures, and increase polymer types and properties [97]. Single-site catalysts based on group 4 metals, e.g., zirconium and titanium, are activated with methylaluminoxane (and its modified forms) or borate activators to generate uniform active sites, resulting in narrow MWD and controlled stereocontrol. Catalysts such as rac-Et(Ind)_2_ZrCl_2_ produce isotactic PP with highly regular helical sequences, whereas fluorenyl-cyclopentadienyl produces syndiotactic PP. Industrially, these catalysts are used mainly in bulk (slurry/loop) and gas-phase reactors, with solution processes reserved for specialty grades that require tight molecular weight and tacticity. ZN catalysts account for more than 97% of global PP manufacturing, and recent innovations have focused on improving efficiency, sustainability, and material performance.

#### 2.3.2. PP Alternative Synthesis Routes

Several alternative synthesis routes have emerged, aiming to expand microstructure control, such as tacticity, comonomer tolerance, and programmed long-chain branching, and to reduce lifecycle emissions by integrating bio/circular feedstocks and partial electrification upstream of propylene supply. Among the alternative routes, the use of different catalysts stands out, and research involving these compounds is growing and highly innovative.

Beyond classical metallocene and late transition-metal platforms, e.g., Ni, Fe, Co, and Pd, post-metallocene platforms, e.g., FI phenoxy–imine and constrained geometry non-metallocenes, enable functional-group tolerance, improved comonomer response, and programmable stereocontrol. Zou et al. (2023) [98] reported that Ni catalysts have high potential for industrial applications and investigated their behavior considering hydrogen and ionic bond anchoring, coanchoring, and ionic cluster formation. Authors reported excellent morphology control, enabling the development of high-performance polyolefins with potential applications across different fields [98]. Alternative routes are of great interest for the development of new catalysts, as they expand the catalyst design space, enabling precise control over comonomer response, stereoregularity, long-chain branching, and molecular-weight distribution. By tailoring ligand symmetry and activator systems, and by engineering supporting catalysts and reactor staging, producers can fine-tune melt index, crystallization behavior, optics, and impact–stiffness balance to match target applications.

A recent catalytic concept is dual-chain growth, in which a single metal center propagates two chains that couple via a diene to form long-chain branches in one catalytic sequence [99]. This approach can deliver long-chain branching PP in slurry/gas reactors, enhancing melt strength and bubble stability without the need for autoclaves. Bergstra et al. (2022) prepared a review of the Borstar PP hybrid polymerization process, a method that aligns technology, catalytic systems, and polymer design [100]. The authors noted that using a single-metal-center system in this process is challenging due to its high cost and operational constraints. Companies like Borealis, Sinopec, Kingfa, and SABIC have developed proprietary catalytic systems that enhance polymerization efficiency and product purity. These systems are BPA-free and support high-performance applications [101]. In addition to catalyst changes, another possibility is to integrate heterophasic PP into the reactor. Using two-stage processes, such as gas–gas, loop–gas, or a staged gas-phase, allows the development of a rubber phase inside the reactor, directly dispersed in PP, resulting in heterophasic impact PP with composition and MWD control. Valaei and Bartke (2022) reported a two-step polymerization, in which the first step involves the bulk-phase polymerization of propane, and the second step is the gas-phase copolymerization of propene and ethylene [99]. Zhao et al. (2024) [102] evaluated multicomponent PP systems obtained by in-reactor polymerization to increase the rubber content in isotactic polypropylene/ethylene–propylene rubber (iPP/EPR). Authors reported a material with superior mechanical properties due to the rubber phase distribution and elasticity, resulting in an IPP/EPR alloy with potential application in various industrial sectors [102].

The industry is shifting toward bio-based PP derived from renewable resources, such as sugarcane and corn. These processes reduce carbon emissions and eliminate BPA exposure, aligning with global sustainability goals [103]. In this way, bio-naphtha and bio-propane or propene can feed conventional steam cracking, yielding drop-in propylene for PP synthesis with certified mass-balance or segregated tracking. Bio-PP has been small until now, but strategically important. The Nova-Institute’s 2024 update notes that PP made from bio-naphtha is now established and expanding, although the institute revised its accounting to reflect that only ~10% of reported PP output is verified bio-based content and ~90% is mass-balance/free attribution under certification schemes [104]. In 2023, bio-based PP accounted for <1% of total bio-based polymer production, underscoring its relative early stage compared to PLA, PA, and others. Still, capacity is projected to grow strongly through 2028 (~35% across selected families).

Taken together, alternative PP routes coalesce around a pragmatic synthesis template, including broadening microstructure control with advanced catalysts across the same bulk/slurry and gas-phase programs, designing in-reactor morphology to replace part of the downstream compounding, and decarbonizing the propylene slate upstream via bio/circular feedstocks and partial electrification without altering polymerization chemistry. These pathways preserve rate, tacticity control, and comonomer response, while expanding the design space. Importantly, all remain BPA-free by construction, aligning with the article’s safety scope and regulatory expectations. Additionally, the transition from laboratory-scale catalyst development to industrial implementation remains challenging due to cost, sensitivity to impurities, and process complexity. Consequently, conventional ZN systems continue to dominate industrial production.

#### 2.3.3. Recent Breakthroughs (2023–2025)

Over the past two years, advances in PP synthesis have clustered around three levers: (i) catalyst innovation beyond classical ZN and group 4 metallocenes to late-transition-metal and post-metallocene families that widen stereocontrol and comonomer tolerance, (ii) reactor-level integration that programs morphology in-reactor and MWD, and (iii) platform knowledge on activator/cocatalyst effects and support engineering that improves active-site quality, comonomer response, and heat/mass transfer. Together, these developments extend the PP design space, including tacticity (iPP/sPP), controlled or programmed long-chain branching (LCB), and heterophasic architectures.

New Co/Fe/Ni/Pd catalyst families and post-metallocene ligand frameworks deliver improved functional-group tolerance and comonomer response, targeting propylene (co)polymerization regimes that were less selective with early-metal systems. Sun et al. (2024) [105] used the hybrid steric ligand modification strategy to increase polymerization activity and branching degree using α-diimine catalysts. The developed material exhibited a high molecular weight and a significantly high activity level, and the 4-tert-butylphenyl catalyst showed outstanding behavior [105]. Recent work highlights steric-tuning strategies around late-metal centers that unlock activity/selectivity and provide MWD and microstructure control. A clear trend is heterogenization (supporting homogeneous catalysts) for conventional slurry/gas assets.

Modeling and plant-oriented studies of integrated trains (tubular loop plus multizone gas-phase, or loop–gas) demonstrate how stage-specific feeds, e.g., ethylene in the second stage, per-stage hydrogen, and residence–time distribution can program EP–rubber fraction and composition alongside bimodal/trimodal MWD. This replaces the downstream compounding part with an in-reactor morphology design, improving interphase adhesion and the impact–stiffness trade-off at lower carbon and cost footprints. Manuyko et al. (2022) [106] investigated PP polymerization in a loop reactor on a Ti-Mn catalyst using modeling. The authors reported that PP molecular weight is regulated by hydrogen consumption, and polymerization speed increases by 30–40% when the hydrogen concentration increases from 0.5 to 2.5% [106]. Updated reviews, such as those prepared by Li et al. (2024), connect chain architecture and phase composition in propylene-based elastomers to viscoelastic and impact performance, useful design rules for propylene copolymerization, and heterophasic iPP grades, in which the elastomeric phase is built in the reactor rather than post-blended [107]. In addition to this, high-throughput experimentation (HTE), inline analytics, and data-driven modeling compress the ligand/activator and performance cycle, improving reproducibility and enabling faster translation from discovery to pilot [87].

### 2.4. Polystyrene (PS)

Polystyrene (PS), CAS number 9003-53-6, is a high-volume thermoplastic derived from aromatic styrene monomers and offers high processing versatility, including injection, extrusion, and thermoforming, as well as suspension/emulsion routes. Different families can be obtained from this common polymer, including the traditional PS (GPPS), valued for optical clarity and stiffness, high-impact PS (HIPS), toughened by a rubber phase for appliance housings and packaging, and foamed PS (EPS/XPS), used for thermal insulation and protective packaging [108]. In addition to this, syndiotactic PS (sPS), obtained via single-site catalysis, exhibits high crystallinity and improved thermal/chemical resistance compared to the traditional one [109].

**Figure 4:**
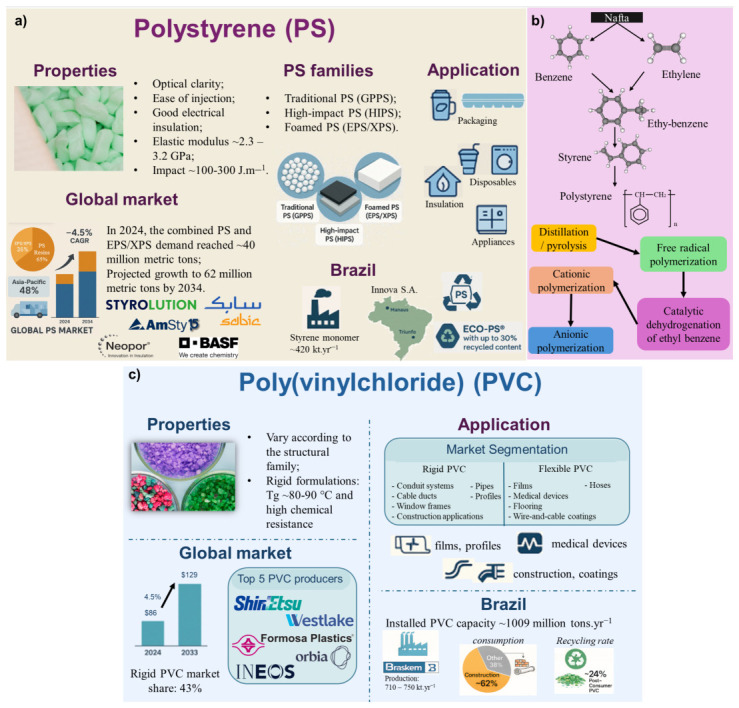
(**a**) Polystyrene (PS) overview: properties, global market, main producing companies, applications, and Brazil’s main numbers, and (**b**) simplified PS synthesis route and common synthesis methods. (**c**) Polyvinyl chloride (PVC) overview: properties, applications, global, and Brazilian market.

The global PS market in 2024/2025 is commonly reported in two scopes: i. PS resins, and ii. combined PS + EPS/XPS. Recent industry reports state that the total PS market (PS + EPS/XPS) reached ~40 Mt in 2024, with projections of ~62 Mt by 2034 (~4.5% CAGR 2025–2034), reflecting continued demand in packaging, appliances/electronics housings, and building insulation. Figure 4a summarizes the main information described in this section.

The PS resin type is the second-largest segment, with a ~35% market share in 2024, while foams dominate the global market, with 60% of the total [110]. Considering the expanded PS market size, the expected growth is 3.0%, with an actual volume (2025) of ~13 Mt and expected volume of ~15 Mt. According to the report published by Mordor Intelligence, the EPS market is consolidated, with the industry leaders including INEOS Styrolution (the largest global styrenics supplier, Germany), BASF (Germany), BEWi (Norway), Alpek S.A.B. de C.V. (Mexico), SABIC (Saudi Arabia), and Synthos (Poland). In 2024, BASF enhanced the styrene value chain by increasing production capacity at Neopor, Ludwigshafen by 50,000 kt.year^−1^ annually [111]. Other global companies, considering all PS families, are TotalEnergies (France), Americas Styrenics (AmSty, USA), CHIMEI (Taiwan), PS Japan Corporation, Shanghai SECCO, Versalis (ENI, Italy), Supreme Petrochem (India), and Trinseo (multiple countries across North America, Asia Pacific, and Europe) [112].

#### 2.4.1. PS Common Synthesis Routes

Historically, PS was first developed by Dow Chemical Company in the 1930s, while I. G. Farben developed the continuous tower process in Germany [113]. Its synthesis occurs via styrene monomer, produced by the dehydrogenation of ethylbenzene, which is derived from benzene and ethylene via Friedel–Crafts catalysis with aluminum chloride or a gas-phase process using zeolites. Figure 4b illustrates styrene synthesis and the PS polymerization process, including the raw materials. The challenges included investigating different reactors for continuous processes, including towers and tube tanks with agitation and batch stirred tanks. However, the incomplete conversion did not fully convert the monomer into a solid polymer, and devolatilization processes were studied [114]. Some constraints were encountered during commercial process selection, such as heat, since PS degrades rapidly at temperatures above 250 °C, and the alternative was the continuous solution process with agitation. Free-radical polymerization remains the dominant industrial route due to its operational simplicity, scalability, and economic viability, enabling the production of general-purpose PS (GPPS), expanded PS (EPS), and high-impact PS (HIPS). According to Sheng et al. (2004), polymerization can be induced by heat or peroxides [115], following first-order kinetics at high styrene concentrations, while chain termination typically proceeds through radical recombination or disproportionation mechanisms [116]. As a limitation, poor control over dispersity and polymer architecture can be mentioned [117]. Styrene can be polymerized via different mechanisms, including radical, cationic, anionic, and metallocene catalysis, and using bulk, suspension, solution, and emulsion methods; descriptions are presented in the Supplementary Material. Table 1 compares the main industrial and academic/laboratory synthesis routes for polystyrene, highlighting their advantages, limitations, and applications.

Anionic polymerization is a route used at laboratory and semi-industrial scales, allowing precise control of molecular weight and tacticity (e.g., syndiotactic PS), but requiring strictly anhydrous, impurity-free conditions. The most common mechanism for this route involves lithium as a counterion and nonpolar solvent [118]. Jozaghkar et al. (2022) [119] investigated PS anionic polymerization at 45 °C using sec–butyllithium and cyclohexane as an initiator and solvent, respectively. The authors reported a propagation rate constant of 0.65 L·mol^−1^·s^−1^ and proved that this low-temperature method is adequate for PS synthesis [119]. From a process view, suspension and emulsion polymerization, applied mainly for EPS production, generate spherical particles that can be expanded later. The obtained polymer exhibits excellent heat transfer, which is crucial for large-scale processes [117].

Table 1 provides a comparative overview of the main PS polymerization routes; an industrial decision requires a deeper understanding of the operational constraints and process conditions associated with each route. In this case, factors such as heat removal efficiency, reaction kinetics, devolatilization requirement, impurity tolerance, and reactor configuration influence polymer quality, energy consumption, and economic feasibility. To complement the industrial information, Table 2 summarizes key technical industrial processes parameters, including initiator systems, temperature and pressure ranges, monomer conversion, and typical equipment used.

#### 2.4.2. PS Alternative Synthesis Routes

Recent advances in polymer science have intensified research into alternative synthesis routes for PS, primarily driven by environmental pressures, regulatory scrutiny of styrene emissions, and the growing demand for polymers with enhanced recyclability or controlled degradability. While conventional free-radical routes remain dominant industrially, several emerging strategies aim to modify PS structure at the molecular level or redesign its synthesis pathway to enable chemical deconstruction, stimuli-triggered depolymerization, or improved circularity. Advances in controlled radical polymerizations, such as ATRP, RAFT, and NMP, have enabled the synthesis of PS with well-defined block sequences, narrow dispersity, and tailored end groups, thereby improving compatibility with elastomers, polar polymers, nanoparticles, and functional fillers [116,120,121]. These methods are particularly attractive for generating PS-based block copolymers, nanostructured domains, compatibilizers, and advanced electronic or biomedical materials [122]. Although not yet dominant in large-scale PS manufacturing, these technologies provide molecular design capabilities unattainable via classical radical processes.

Stereoregulation strategies using anionic polymerization or metallocene catalysis have enabled the synthesis of sPS, characterized by high crystallinity, improved thermal resistance, and superior chemical stability [123]. Coordination catalysts, such as fluorenyl-based metallocenes, have demonstrated high stereoselectivity and productivity, expanding the scope of PS for engineering and high-performance applications [124]. In parallel, alternative monomer activation routes, including cationic polymerization, charge-transfer complex polymerization, and photo-initiated polymerizations, have been investigated to reduce energy consumption, achieve selective functionalization, and enable milder processing environments. Photo-ATRP and photoiniferter methods, in particular, have shown promise for oxygen-tolerant and energy-efficient PS synthesis [125].

More recently, environmental concerns have driven the emergence of degradable and circular PS design strategies. A breakthrough reported by Li et al. (2025) introduced an ultra-high-molecular-weight PS, containing degradable linkages covalently incorporated into the polymer backbone [125]. This approach leverages comonomers bearing cleavable units, such as acetal, thioacetal, carbonate, or photolabile motifs, that maintain PS-like properties while enabling triggered chain scission under specific conditions (heat, UV light, acid/base catalysis). This concept represents a promising pathway toward new generation styrene materials with reduced environmental persistence and potentially improved recyclability via controlled depolymerization. Overall, these emerging synthesis strategies extend the PS design space beyond commodity GPPS, HIPS, and EPS, enabling tailored architecture, advanced functionalities, and exploration pathways toward circular styrenic materials [126,127]. As described for other polymers, alternative approaches are limited by high cost, process complexity, and scalability, thereby restricting their industrial adoption. As such, conventional free-radical polymerization continues to dominate large-scale PS production.

#### 2.4.3. Recent Breakthroughs

Polyvinyl chloride (PVC), CAS number 9002-86-2, is derived from the polymerization of vinyl chloride monomer. PVC offers high processing versatility, including extrusion, injection molding, blow molding, coating, and plastisol/organosol methods, enabling a wide range of industrial products [128]. Different structural families are derived from the same polymer: primarily rigid PVC for profiles, pipes, and fittings due to its rigidity, chemical resistance, and dimensional stability, and plasticized PVC for electrical cables, flexible films, hoses, and coatings [129,130]. Due to the diversity of formulations, which vary in impact modifiers, mineral fillers, thermal stabilizers (Ca/Zn, Sn, etc.), plasticizers, and lubricants, the properties of PVC vary widely. In rigid formulations, a Tg of 70–80 °C, an elastic modulus of 2.4–4.0 GPa, high chemical resistance, self-extinguishing properties due to the chlorine content, and excellent extrusion processability are observed. In flexible formulations, PVC exhibits high elongation and toughness, with fine control of flexibility and hardness [131].

According to the Global Growth Insights report, the global PVC market was valued at ~US$ 86 million in 2024 and is projected to reach US$ 129 million by 2033, with a CAGR of 4.5%. Similar to other polymers described in this work, Asia-Pacific dominates the global market (34% market share), with China accounting for ~45% of global demand [132]. The European PVC market is considered highly lucrative, with a ~24% share in 2023, mainly to meet construction demand [133]. The rigid PVC segment dominates the global market, accounting for over 60% of total demand, with particular applications in pipes, profiles, conduct systems, cable ducts, and window frames. In contrast, flexible PVC continues to expand, especially in films, medical devices, flooring, hoses, and wire-and-cable coatings, representing approximately 40% of the total market [132]. These data reflect market segmentation by application and highlight the predominance of rigid PVC in construction and infrastructure, while flexible PVC serves growing industrial and consumer niches.

The global PVC industry is highly consolidated, with the five largest producers controlling ~43% of the world’s installed capacity. Shin-Etsu Chemical Co. (Japan) remains the dominant global supplier, leveraging fully integrated chlor–alkali operations, advanced energy-efficient production units, and geographically diversified assets across Asia and North America. The other top companies are Westlake Chemical (USA), Formosa Plastics Corporation (Taiwan/USA), Orbia/Mexichem (Mexico/Brazil), and INEOS (UK) [133]. These groups operate global hubs capable of producing commodity PVC grades, as well as specialty formulations for medical devices, high-performance pipes, films, and profiles. The companies Westlake and Formosa follow closely, leveraging large-scale ethylene, VCM, and PVC production hubs in North America and Asia [132]. In 2024, Formosa Co. announced a major expansion of its production site in Louisiana, USA, to meet growing global PVC demand. Additionally, Westlake announced an intention to expand PVC production [134]. In 2025, Orbia introduced a PVC recycling initiative to repurpose this plastic for various applications [133]. Figure 4c summarizes the main information described in this section.

### 2.5. Polyvinyl Chloride

#### 2.5.1. PVC Common Synthesis Routes

**Figure 5:**
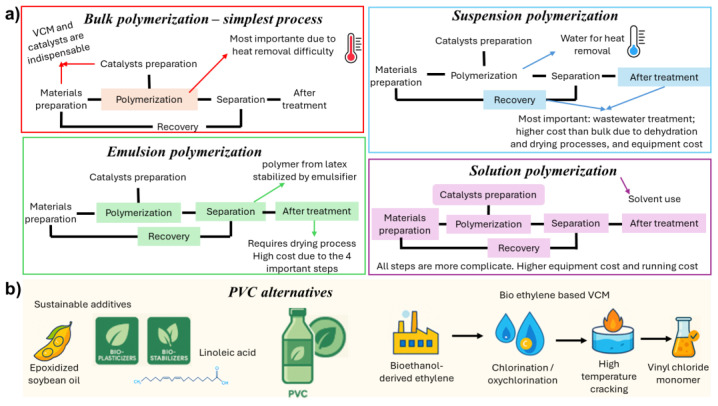
(**a**) Overview of PVC industrial polymerization routes (suspension, emulsion, bulk, and solution), and (**b**) summary of sustainable PVC alternatives, including bio-based plasticizers and stabilizers, and bio-ethylene-to-VCM pathway derived from bioethanol via chlorination/oxychlorination and high-temperature cracking.

PVC industrial development began in the early 20th century, with large-scale commercialization led by BFGoodrich in 1926, following earlier discoveries of vinyl chloride polymerization by Baumann in 1872 and Klatte in 1912 [135]. Modern PVC production relies on vinyl chloride monomer (VCM), a colorless gas under normal temperature and pressure. PVC is synthesized via free-radical polymerization of VCM. Common production processes include bulk, suspension, emulsion, and solution polymerization, as illustrated in Figure 5a. Table 3 summarizes the main advantages, limitations, and applications of these methods, as well as the main processing conditions of each process [136].

Industrial PVC production is dominated by suspension polymerization (PVC-S), which accounts for ~80–85% of global output and produces granular resins suitable for rigid applications such as pipes, profiles, and fittings [37]. Emulsion polymerization (PVC-E) accounts for ~10–12% of global production and yields fine-particle latexes essential for coatings, flooring, gloves, synthetic leather, and flexible films. Common routes face challenges related to VCM toxicity, polymerization autoclave fouling, and strict residual monomer regulations (<1 ppm in many markets), which require advanced stripping operations and high-vacuum devolatilization systems. Suspension PVC is inherently amorphous and brittle, requiring plasticizers (e.g., phthalates, adipates, and citrates) for flexible applications. Emulsion PVC, due to its smaller particle size, forms plastisols with controlled rheology and faster fusion rates. The emulsion process allows the production of fine-particle latexes, enabling the production of resins with high surface area and optimized gelation behavior. These characteristics are essential for flooring, synthetic leather, gloves, adhesives, sealants, and high-performance coatings. As in PS, industrial suspension processes benefit from improved agitation systems, optimized initiator dosing, and enhanced heat-transfer strategies [137,138].

Taken together, industrial PVC synthesis integrates mature free-radical routes with continuous improvements in reactor design, process control, and resin tailoring. Ongoing innovations, particularly in heat-transfer enhancement, fouling mitigation, particle-morphology engineering, and residual-VCM removal, are essential to meet increasingly stringent regulatory and performance requirements.

#### 2.5.2. PVC Alternative Synthesis Routes

Unlike PS or polyolefins, where alternative synthetic routes may involve fundamentally different polymerization mechanisms, research on PVC generally focuses on sustainable formulations and greener feedstocks rather than altering the intrinsic radical VCM polymerization of vinyl chloride monomer. Thus, innovation is concentrated on more sustainable additives, stabilizer systems, and recycling pathways that can reduce environmental and toxicological impacts while maintaining compatibility with established suspension and emulsion production technologies [139,140].

A major research front involves developing bio-based plasticizers to reduce the toxicity, migration, and volatility associated with conventional phthalates. Prominent examples include epoxidized soybean oil (ESO) derivatives, esters derived from linoleic acid and cinnamyl alcohol, which exhibit improved migration resistance and plasticization efficiency [141]. Burns et al. (2023) used ESO as a bio-plasticizer and reported good plasticizing performance, with PVC formulations showing higher elongation and tensile strength compared to those containing conventional plasticizers [142]. In parallel, efforts to develop green thermal stabilizers aim to eliminate historical reliance on lead-based systems. Modern alternatives, particularly calcium–zinc, calcium–organic, and fully organic stabilizer packages, offer improved recyclability and eliminate heavy-metal contamination throughout the product life cycle. Wen et al. (2015) prepared hydrocalumite and mixed them with PVC resin, resulting in superior long-term and initial thermal stability for the composites [143]. Yang et al. (2023) [144] studied the effects of epoxidized cardanol ester as a plasticizer and thermal stabilizer. The authors reported the potential applicability of the developed PVC films, owing to the excellent plasticizing effect and high conformational mobility of the polymer with cardanol–myristate, in addition to the high initial thermal decomposition temperature of 314 °C [144].

Recent advances in bio-based plasticizers have focused on improving migration resistance, thermal stability, and compatibility with PVC matrices, aiming to overcome the limitations of conventional phthalates. In addition to epoxidized vegetable oils, new classes of plasticizers derived from renewable feedstocks, such as citrate esters [145], triphenylacetic glyceroate [146], lauric acid ester–amide [147], isosorbide derivatives [148], and cardanol-based compounds, have demonstrated promising performance. These materials often exhibit lower volatility and reduced toxicity while maintaining, or even enhancing, flexibility and elongation at break. For instance, cardanol-derived plasticizers have shown improved permanence and oxidative stability due to their aromatic structure [149], whereas citrate-based systems are widely recognized for their suitability in food-contact and medical applications [150].

More recent studies have also explored multifunctional plasticizers that act as stabilizers, reducing the need for additional additives and improving formulation efficiency. Despite these advances, challenges related to long-term durability, extraction resistance, and cost competitiveness remain significant barriers to large-scale industrial adoption. Therefore, ongoing research is increasingly focused on optimizing molecular design and balancing performance, safety, and economic feasibility.

A promising long-term alternative is the development of bio-ethylene-based VCM pathways, in which ethylene derived from bioethanol is chlorinated or oxychlorinated to produce EDC, followed by high-temperature cracking to produce VCM [151]. This route maintains full chemical equivalence with petrochemical VCM, enabling direct integration into existing PVC assets without structural modifications. Recent assessments highlight that bio-ethylene production via second-generation bioethanol and catalytic dehydration has reached high technological maturity and can significantly reduce cradle-to-gate carbon footprints [152] Nevertheless, despite its technical feasibility and compatibility with current chlor-vinyl infrastructure, commercial adoption remains limited, primarily due to elevated production costs, the availability of sustainable ethanol, and the scale required to compete with established ethylene/EDC/VCM chains [153]. Even so, bio-VCM continues to be recognized as a strategic pathway for medium- and long-term PVC decarbonization sector, especially in regions with strong biomass supply chains and established bioethanol industries [154]. Figure 5b shows a representation of PVC alternatives.

#### 2.5.3. Recent Breakthroughs

Recent advancements in PVC technology have focused primarily on enhancing process safety, energy efficiency, and environmental performance, while preserving the well-established radical VCM polymerization mechanism. One important front is processing intensification, particularly the transition from traditional batch operations to continuous suspension polymerization, which has demonstrated improvements in thermal management, operational stability, and intrinsic process safety. Continuous systems also enable more precise control of particle morphology and heat removal, which are critical parameters in PVC production, and offer potential reductions in emissions and overall energy consumption. Complementary to these efforts, innovations in direct water-recycling strategies have emerged to reduce environmental impacts. Recent studies integrating water reuse into suspension polymerization indicate that freshwater consumption and wastewater generation can be significantly reduced without compromising resin quality or processing performance. These developments align with broader industrial targets for resource efficiency and circular manufacturing.

Advances in greener formulation components represent a major technological trend. Replacement of traditional additives, e.g., lead-based stabilizers, has accelerated with the adoption of calcium–zinc or organic stabilizer systems. In parallel, bio-based plasticizers derived from renewable feedstocks, such as modified vegetable oils and fatty acid esters, have demonstrated improved toxicological profiles, reduced migration, and high compatibility with PVC matrices. Together, these additive innovations contribute to safer formulations while facilitating recyclability, especially in flexible PVC applications.

Research has also expanded into functionalized PVC composites and improved control of molecular architecture. The incorporation of nanofillers, such as graphene, silica, and layered materials, has been shown to enhance mechanical integrity, flame retardancy, and barrier performance, broadening the applicability of PVC in high-performance construction, electrical, and protective-coating sectors. Additionally, progress in controlled radical processes enables more tailored molecular-weight distributions and composite structures, offering pathways for advanced material design without altering the fundamental polymerization mechanism. Importantly, across all industrial routes and initiator systems, bisphenols (e.g., BPA) are neither reagents nor byproducts of PVC synthesis; therefore, any detected bisphenol contamination originates exclusively from external contact or post-processing exposure.

Despite these advances, the large-scale implementation of chemical recycling and upcycling technologies remains heavily dependent on the availability of well-sorted, high-purity plastic waste streams. These limitations are highlighted by the extremely low rate of chemical recycling, which accounted for only 1% of total German recycling in 2018 [155,156]. Alrazen et al. (2025) reported that chemical recycling depends on catalyst type and operating conditions, and the heterogeneous nature of plastic waste poses a challenge due to the variety of raw materials and contaminants, requiring pre-treatment technologies [157]. According to Chidara et al. (2025), chemical recycling needs to focus on developing new methods, enhancing regulatory compliance, and intensifying international collaboration [129]. The primary barriers described include inadequate waste collection infrastructure, varying recycling protocols, and consumer resistance to the use of recycled PVC [158].

Table 4 provides a comparative overview of the five major commodity polymers—PET, PE, PP, PS, and PVC—covering their dominant processing and recycling methods, typical operating parameters (temperature and pressure), and the catalysts used. It also highlights the current industrial adoption rates of each method, showing that mechanical recycling dominates PET, Phillips and Ziegler–Natta catalysts remain the backbone of PE and PP production, free-radical polymerization overwhelmingly drives PS manufacture, and suspension polymerization is the main route for PVC. Together, these data illustrate both the technical diversity of polymer processing and the market realities of how industries apply these methods today. In sum, regarding PET, mechanical recycling dominates (~70%), but chemical recycling is growing (~25%) as contamination challenges rise. In the case of PE, Phillips catalyst processes still lead (~60%), but metallocene adoption (~30%) is expanding due to better property control. PP manufactured via the Ziegler–Natta route remains the industry standard (~85–90%), while metallocene catalysts are gaining niche applications. PS free-radical polymerization is overwhelmingly dominant (~95%), while controlled radical methods are mainly used in research and specialty products. Finally, PVC suspension polymerization is the main route (~80–85%), while emulsion (~10–15%) serves specialty applications; recycling remains marginal (<5%).

## 3. Regulatory Framework and Global Initiatives

### 3.1. The Evolution of Legislation on BPA and Additives in Plastics in the EU, United States, and Brazil

Regulatory frameworks governing bisphenol A (BPA) and other critical additives in plastics have evolved substantially across the European Union, the United States, and Brazil, reflecting advances in toxicological evidence, heightened public awareness, and the global push for safer materials. The European Union has implemented the most comprehensive and restrictive measures, culminating in Regulation (EU) 2024/3190, which harmonizes and tightens controls within the EU internal market on BPA and structurally related bisphenols across plastics, varnishes, and coatings intended for food contact. This regulation updates the technical annexes of Regulation (EU) No. 10/2011 and repeals Regulation (EU) 2018/213 by substantially lowering migration limits, in practice prohibiting BPA in food-contact plastics, and broadening the scope of regulated substances to strengthen consumer protection [174]. Importantly, current regulatory approaches recognize that the absence of specific substances, such as BPA, in polymer synthesis does not fully define material safety. Instead, safety assessments increasingly consider complete formulation, including additives, non-intentionally added substances (NIAS), degradation products, and migration potential under realistic conditions of use.

In the United States, oversight is primarily conducted through FDA toxicological evaluations rather than through a unified migration-limit framework. BPA was banned from baby bottles and sippy cups in 2012, but the FDA continues to evaluate its use in broader food-contact applications through case-specific risk assessments [175]. Brazil follows a similar risk-based structure: ANVISA’s RDC No. 41/2011 prohibits BPA in baby bottles, while a family of complementary RDCs regulates monomers, additives, migration testing, and analytical conformity for food-contact plastics [176]. Together, these frameworks illustrate a trend toward increasing regulatory convergence, driven by scientific reassessment and alignment with international safety guidelines.

### 3.2. Standards for Food-Contact Materials and Contaminant Migration Limits

Across regulatory jurisdictions, food-contact materials are required to ensure a high level of consumer protection by preventing risks to human health and avoiding unacceptable changes in food composition, organoleptic properties, or quality. This principle is established in the EU by Framework Regulation (EC) No. 1935/2004 and further specified by Commission Regulation (EU) No. 10/2011, which sets an overall migration limit (OML) of 60 mg·kg^−1^ of food and establishes specific migration limits (SMLs) or other restrictions for more than one thousand authorized substances. These include substances subject to numerical SMLs, as well as substances for which migration is no longer permitted under current EU regulations, such as bisphenol A (BPA). Brazilian regulations adopt the same risk-based framework by prescribing overall and specific migration testing, standardized food simulants, defined time–temperature exposure conditions, and validated analytical methods. In contrast, the U.S. FDA evaluates food-contact substances through toxicological review and dietary exposure assessment on a case-by-case basis, rather than through a unified overall migration limit.

Growing regulatory pressure and global sustainability commitments have accelerated research into safer, recyclable plastic materials. Policy frameworks, such as the OECD’s initiatives for sustainable plastic design and the EU’s Circular Economy Action Plan, promote the development of non-toxic additives, improved material traceability, and recyclable or bio-based polymer systems. Brazil and the United States similarly support innovation through public funding programs, fiscal incentives, and industry–academia collaborations focused on safer stabilizers, BPA-free additive packages, bio-based feedstocks, and advanced recycling technologies. These mechanisms demonstrate how regulatory evolution not only constrains hazardous substances but also drives material innovation and accelerates the adoption of circular-economy strategies.

## 4. Future Perspectives and Final Remarks

The future of conventional plastics is being shaped by advances in catalysis, feedstock diversification, safety-by-design strategies, and circular-economy technologies. Polymers, such as PET, PE, PP, PS, and PVC, are already produced in free-bisphenol synthesis and processing, as presented in this review, and are well-described in the literature. These polymers exhibit well-established safety profiles when manufactured and processed under regulated conditions; nevertheless, emerging societal, regulatory, and environmental demands are pushing the sector toward deeper transformation. Table 5 presents a comparative analysis of all described polymers, highlighting key parameters that characterize their sustainability and performance. The information presented in this table is essential to illustrate the potential of all these commodity polymers.

The carbon footprint values reported in Table 5 are derived from peer-reviewed life-cycle assessment (LCA) studies and industry datasets. To ensure consistency and transparency, some methodological choices were adopted. Regarding system boundaries, unless otherwise specified, values are reported on a cradle-to-gate basis, encompassing raw material extraction, monomer synthesis, and polymer production. For studies that include recycling, use phase, or disposal, results are reported as cradle-to-grave and explicitly noted in references. For the functional unit definition, all values were normalized to 1 kg of polymer produced. The selected studies represent widely cited benchmarks in the scientific literature and industry reports. Reported ranges (e.g., PET 2.1–2.5 kg CO_2_ eq/kg) reflect variability across different production routes, energy mixes, and geographic contexts. Finally, where available, LCA studies of bio-based feedstocks (e.g., bio-MEG for PET, and bio-ethylene for PE) were included to highlight potential reductions in carbon intensity.

In this context, several innovative trajectories are expected to redefine polymer production, conversion, and end-of-life management over the next decade. However, to bridge the gap between conceptual promise and industrial reality, it is essential to evaluate the current maturity of these advancements. As summarized in Table 6, the Technology Readiness Level (TRL) for these innovations varies significantly, ranging from established TRL 9 commercial processes to emerging TRL 4–6 pilot-scale developments. A central trend is catalysis innovation, driven by the need for higher efficiency, lower environmental impacts, and improved control over molecular architecture. Advances in late-transition-metal catalysis, hybrid metallocene/heterogeneous systems, and data-driven catalyst design are enabling fine-tuning of molecular weight distribution, stereoregularity, comonomer incorporation, and branching. These developments support the production of customized resins that deliver enhanced mechanical strength, barrier performance, thermal stability, or recyclability, while simultaneously reducing energy consumption and by-product formation. Despite these advancements, the industrial scale-up of late-transition-metal and hybrid systems remains challenging. These catalysts are notoriously sensitive to polar impurities and moisture, often leading to rapid catalyst deactivation or ‘poisoning’. Achieving the required feedstock purity (at ppb levels) introduces significant pretreatment costs, which can offset the economic benefits of improved molecular control. These limitations are reflected in the current TRL (Table 6), where many emerging recycling and catalytic processes are still transitioning from pilot-scale validation to full-scale commercial deployment.

At the same time, the increasing availability of biomass-derived intermediates and CO_2_-based building blocks is expanding opportunities for integrating renewable feedstocks into conventional polymer pathways. Biological and electrochemical routes to ethylene, paraxylene, ethylene glycol, and acrylic monomers continue to advance, bringing the prospect of “drop-in” bio-based PET, PE, and PP with reduced life-cycle emissions. Progress in catalytic pyrolysis, solvolysis, and electrochemical depolymerization also points to the emergence of hybrid systems that combine fossil and renewable carbon sources with high-purity recycling loops. Another promising frontier lies in the evolution of circular technologies. Chemical recycling is transitioning from conceptual demonstration to industrial deployment, a shift reflected in its current TRL 7–8 status (Table 6), indicating ongoing efforts to navigate significant engineering and economic scalability hurdles. The advances are supported by reactor innovations, improved depolymerization catalysts, and integration with mechanical recycling to create high-quality secondary feedstocks. The use of digital traceability tools, including polymer tagging, AI-enabled sorting, and blockchain-based identity systems, promises to increase material purity, prevent cross-contamination with BPA-containing materials, particularly from legacy polycarbonate streams, and strengthen supply-chain transparency, particularly for food-contact applications.

Alongside these advances in catalysis, feedstock diversification, and circular-economy strategies, nanomaterials have emerged as important enablers in polymer science and engineering. The incorporation of nanofillers, such as nanoclays, graphene, carbon nanotubes, and metal–oxide nanoparticles, has been increasingly explored to enhance the mechanical performance, barrier properties, thermal stability, and flame retardancy of commodity polymers, including PET, PE, PP, PS, and PVC. Beyond property enhancement, nanomaterials also play a significant role in processing and recycling technologies, acting as nucleating agents, reinforcing phases, rheology modifiers, and catalytic or catalyst-support platforms in depolymerization and upcycling processes. Musthafa and Mandal (2025) developed a method for the degradation of PE, PP, PVC, and PET using NiO nanoparticles (NPs) [187]. The authors reported that the NPs increased photocatalytic activity, and PET films containing 2% NiO showed 42% degradation in 30 days under visible light. Boruah and Lopez-Ruiz (2024) described photocatalysis in the liquid phase for the degradation of PE, PP, and PS using light irradiation [188]. Their review, reporting several works that investigated photocatalytic conversion using different nanoparticles and technologies, is as follows: ZnO nanoroads that facilitate oxidative decomposition of LDPE [189]; Nb_2_O_5_ monolayer nanosheets to degrade PE and PP into CO_2_ and acetic acid [190]; and Co-Ga_2_O_3_ nanosheets to reduce commercial products, such as PP boxes, PE bags, and PET bottles into powders with a size <5 mm [191], among others. Additionally, nanoparticle-enabled catalysis has shown potential to improve reaction efficiency, selectivity, and energy consumption in chemical recycling routes. Selvam et al. (2023) described the use of Zn-based nanoparticles with different morphologies in PET microwave-assisted glycolysis and reported that smaller NPs lead to higher chemical conversion rates due to their higher surface area [192]. Sarwan et al. (2020) synthesized BiOCl as nanoflowers and nanodisks, and prepared PS films using these nanoparticles, the results indicated that, after irradiation, the films were photo-oxidized due to the NPs’ action in generating hydroxyl radicals [193]. These findings highlight the strong potential of nanomaterial-enabled systems to enhance degradation efficiency and enable alternative recycling pathways. However, challenges related to catalyst recovery, scalability, and process integration remain critical barriers for their large-scale industrial implementation.

In parallel, ongoing advances in polymer safety science are transforming the way materials are evaluated and certified. High-resolution mass spectrometry and predictive toxicology models based on machine learning are examples of methods that enable faster identification of contaminants and more rigorous validation of BPA-free claims. These analytical capabilities are aligned with global regulatory movements that increasingly require exhaustive assessment of non-intentionally added substances and improved risk communication mechanisms.

Cross-sector innovation ecosystems involving polymer producers, recyclers, academia, health agencies, and consumer-facing industries are likely to play a decisive role. The harmonization of global standards, shared analytical platforms, and collaborative regulatory frameworks will be crucial for advancing a unified understanding of polymer safety, minimizing misinformation surrounding BPA, and strengthening societal trust in conventional plastics as the industry evolves toward its next technological horizon. These perspectives indicate that the future of conventional polymers will be marked not by their replacement but by their reinvention toward cleaner synthesis routes, safer additive technologies, circular resource flows, and scientifically robust safety verification. Such developments will redefine the role of conventional plastics within sustainable materials engineering while preserving their indispensable value.

The translation of the chemical and technological evidence presented in this review into effective governance requires a multi-stakeholder approach. To bridge the gap between technical reality and public perception, regulatory frameworks must evolve beyond simple bans toward evidence-based material stewardship. Several key policy recommendations can be derived from the integrated chemical, industrial, and communication perspectives discussed throughout this work, such as:

- Standardization of Safety Labeling: Regulatory bodies (such as ANVISA, FDA, or EFSA) should discourage the use of “BPA-Free” claims on polymers that do not utilize bisphenol A in their synthesis (e.g., PET, PE, and PP). Such labeling practices inadvertently sustain consumer misconceptions and “chemophobia” by implying that BPA presence is a variable risk in all plastics.
- Mandatory Digital Traceability: The implementation of digital product passports or blockchain-based tagging systems to ensure the transparency of recycled content. This is critical for preventing cross-contamination from legacy polycarbonate streams into high-purity commodity recycling loops intended for food contact.
- Incentivizing Advanced Recycling (TRL 6–8): Policy frameworks should provide fiscal incentives (tax credits or R&D grants) specifically for chemical recycling technologies. As demonstrated by TRL status, these technologies require institutional support to overcome energy-intensity hurdles and reach the scale necessary for a true circular polymer economy.

By implementing these strategic pathways, policymakers can ensure that innovation in polymer science is matched by regulatory clarity, fostering an environment where safety-by-design and circularity become the standard for the global plastics industry.

## 5. Conclusions

Considering the available literature and the synthesis pathways reviewed, bisphenols, most notably bisphenol A (BPA), are not intrinsic reactants or structural building blocks in the conventional industrial production of PET, PE, PP, PS, and PVC. Polyolefins, such as PE and PP, are produced via coordination- or metallocene-catalyzed polymerization of simple olefin monomers, yielding nonpolar hydrocarbon backbones in which bisphenolic structures are chemically irrelevant. Similarly, PS is obtained through styrene radical polymerization, and PVC from vinyl chloride polymerization. In both cases, the resulting structure–property relationships, such as glass transition temperature, rigidity, and processability, are governed by phenyl or chloro groups rather than by bisphenolic moieties. PET, while an aromatic polyester, is conventionally synthesized from terephthalic acid (or dimethyl terephthalate) and ethylene glycol and thus remains chemically distinct from BPA-based polycarbonates, despite occasional public or regulatory misconceptions.

Beyond clarifying these chemical foundations, this review provides an integrated perspective that connects polymer synthesis, technological innovation, and regulatory frameworks to address a critical gap between scientific evidence and public perception. From an industrial standpoint, advances in catalysis, process intensification, and recycling technologies, including systems currently approaching high technological readiness levels (TRL 7–8), enable more efficient production routes and higher-quality secondary feedstocks. However, challenges related to feedstock purity, sorting infrastructure, and process scalability remain key barriers to the full implementation of circular systems. Importantly, the absence of BPA in polymer synthesis should not be interpreted as a standalone indicator of material safety, as material safety remains dependent on formulation, processing conditions, and real-use scenarios.

Therefore, ensuring the continued safety and sustainability of commodity polymers depends not only on robust chemical design and regulatory compliance, but also on the development of integrated value chains that combine advanced recycling, digital traceability, and improved waste management systems. In this context, reinforcing accurate scientific communication is essential to support evidence-based decision-making, prevent misconceptions about BPA, and guide the responsible evolution of polymer technologies for industrial and societal applications.

## Figures and Tables

**Table 1:** Comparison of polystyrene synthesis routes, their main characteristics, and typical obtained polymers, considering general-purpose polystyrene (GPPS), high-impact polystyrene (HIPS), expanded PS (EPS), syndiotactic PS (sPS), and others.

Synthesis Route	Scale/Use	Typical Use Conditions	Advantages	Limitations	Typical Applications
Free-radical Polymerization (Bulk/Mass)	Industrial	80–150 °C, thermal or peroxides initiation, requires devolatilization	Simple, low cost, robust, high throughput	Poor control of molecular weight and dispersity	GPPS, HIPS
Free-radical Suspension Polymerization	Industrial	Aqueous phase containing stabilizers, bead formation	Good heat transfer, forms EPS spherical beads	Surfactant residues, limited control	EPS, bead resins
Solution Polymerization	Industrial	Aromatic solvents, controlled heat removal	Viscosity control, suitable for continuous processes	Need solvent recovery, low productivity	Specialty PS, copolymers
Emulsion Polymerization	Industrial/pilot	Surfactants, low viscosity	Fine particle size, good heat removal	Need surfactant removal, purity issues	Latex coatings, specialty dispersions
Anionic Polymerization	Laboratory/niche industrial	n-BuLi, anhydrous conditions	Precise control of molecular weight and tacticity, living polymerization	Requires ultra-pure, anhydrous conditions, costly	sPS, block copolymers
Cationic Polymerization	Industrial	AlCl_3_, TiCl_4_, low temperatures	High rates, useful for certain functionalized styrenics	Strong side reaction, poor control	Modified PS, specialty resins
ATRP (Atom Transfer Radical Polymerization)	Academic/R&D	Cu(I)/ligand catalysts	Excellent molecular weight control, versatility	Requires transition-metal catalysts removal	Block copolymers, advanced nanostructures
RAFT (Reversible Addition–Fragmentation Chain Transfer)	Academic/R&D	RAFT agents, mild conditions	Good control, no metals required	Expansive chain-transfer agents, residuals affect properties	Smart biomaterials, biomedical polymers
NMP (Nitroxide-Mediated Polymerization)	Academic/pilot	SG1/TEMPO-based nitroxides	Metal-free, robust	Limited monomer scope, high temperature	Specialty PS derivatives
Charge-Transfer Polymerization (CTP)	Academic/specialized	Electron donor-acceptor complexes	Enables electronic/functional copolymers	Limited scope, complex	Conductive polymers
Metallocene/Coordination Polymerization	Pilot	Single-site catalyst	Produces stereoregular PS	Expensive, moisture-sensitive	High-performance PS

**Table 2:** Comparison of PS industrial synthesis routes considering reaction type, initiators, heat-removal strategies, and industrial challenges.

Process	Reactor Type	Temperature Range	Initiators	Heat-Removal Strategy	Industrial Challenges
Bulk (Mass) Polymerization	Continuous stirred tank (CSTRs), tubular and tower reactors	80–180 °C	Organic peroxides (benzoyl peroxide, dicumyl peroxide), azo compounds (AIBN)	External jackets, internal coils, multistage reactors, viscosity-controlled heat removal	Highly exothermic system, viscosity increase, risk of thermal runaway, styrene degradation above 250 °C, devolatilization requirements
Suspension Polymerization	Stirred tank with water as continuous phase	80–120 °C	Water-soluble initiators (K_2_S_2_O_8_), oil-soluble peroxides, stabilizers	Excellent heat removal by water phase, bead-level heat dissipation	Stabilizer removal, bead size control, residual monomer, foaming agent incorporation
Emulsion Polymerization	Stirred tank reactors using water as the continuous phase, with surfactants and redox or thermal initiators	30–90 °C	Redox initiator systems, surfactants	Excellent heat removal due to aqueous medium	Produces polystyrene latex, residual surfactants and additives limit purity, additional coagulation and purification steps required, therefore, not commonly employed for GPPS production
Anionic Polymerization	Batch reactors under inert atmosphere	−78 °C to room temperature	n-butyllithium, alkyl-lithium initiators	Low heat release due to highly controlled kinetics	Requires ultra-dry, oxygen-free conditions, expensive, not widely industrial for PS

**Table 3:** Comparison of poly(vinyl chloride) synthesis routes and industrial processes characteristics.

**Route**	**Process Description**	**Advantages**	**Limitations**	**Typical Applications**
Suspension Polymerization	VCM dispersed in water with colloids, polymerized in droplets	Dominant route, good heat removal, granular morphology	Batch complexity, stabilizer management	Pipes, profiles, films, cables
Emulsion Polymerization	VCM emulsified with surfactants, latex particle formation	Very fine particles, excellent dispersion	Surfactant residues, higher cost	Plastisols, coatings, flooring, adhesives
Bulk (mass) Polymerization	VCM polymerized in liquid monomer phase without dispersants	High purity resins and simple formulation	Difficult heat removal, limited scalability	Specialty PVC grades
Solution Polymerization	VCM dissolved in organic solvent, controlled radical polymerization	Precise molecular control and niche copolymerization	Solvent recovery, high cost, limited industrial use	Research-scale, specialty copolymers
**Process Route**	**Typical Conditions**	**Initiators**	**Reactor Types**	
Suspension polymerization	40–70 °C, 5–15 bar, aqueous medium	Organic peroxides (e.g., lauroyl peroxide), azo initiators	Stirred tank (batch/semi-batch), baffle/agitated vessels	
Emulsion Polymerization	40–60 °C, 5–10 bar, surfactant-rich aqueous medium	Peroxides, redox systems (e.g., persulfate/Fe^2+^)	High shear stirred tanks, latex handling systems	
Bulk (mass) Polymerization	40–60 °C, 5–10 bar, neat VCM phase	Peroxides, azo compounds	Tubular/loop reactors, stirred tanks	
Solution Polymerization	40–60 °C, moderate pressure, organic solvent	Peroxides, azo initiators	Stirred tank reactors with solvent recovery	

**Table 4:** Polymers, processing methods, parameters, and industry usage.

Polymer	Processing/Recycling Methods	Typical Parameters (Scientific References)	Industry Usage (%) (Market References)
PET (Polyethylene Terephthalate)	- Mechanical recycling (industrial scale) - Chemical recycling: glycolysis, methanolysis, hydrolysis - Upcycling with bio-based monomers	- Glycolysis: 180–240 °C, Zn/Mn acetate [42] - Methanolysis: 180–280 °C, 2–4 MPa [49] - Hydrolysis: 200–250 °C alkaline medium [47] - Upcycling with bio-based monomers [10,38,40,41,43,44]	~70–75% mechanical recycling ~20–25% chemical recycling <5% upcycling [12,159,160,161,162]
PE (Polyethylene)	- Polymerization via Phillips Cr/SiO_2_ catalysts - Metallocene/post-metallocene polymerization - Steam cracking for ethylene feedstock	- Polymerization: 70–110 °C, 1–5 MPa [62,63] - Phillips catalyst Cr/SiO_2_ [64,65,66] - Metallocene: 50–90 °C [67,86] - Steam cracking: 750–850 °C, ~2 bar [69,70,74,75]	~60% Phillips/Cr catalysts ~30% metallocene/post-metallocene [163] ~10% bio-based routes [164]
PP (Polypropylene)	- Ziegler–Natta polymerization with internal donors - Borstar hybrid bulk/gas phase - Homogeneous catalyst elastomers	- Polymerization: 60–90 °C, 1–3 MPa [76,97] - Borstar: 70–90 °C, 2–3 MPa [100] - Isotacticity control via internal donors [76,77,78,80]	~85–90% Ziegler–Natta ~8–10% metallocene <5% homogeneous catalysts [101,165]
PS (Polystyrene)	- Free-radical polymerization (bulk, peroxide initiators) - Living anionic polymerization - RAFT/ATRP controlled radical polymerization	- Free-radical: 100–150 °C [115,116] - Anionic: −78 °C, cyclohexane/n-BuLi [118,119] - ATRP: 60–120 °C [120,124] - RAFT: 70–110 °C [121]	~95% free-radical polymerization [117,166] Living anionc polymerization [108,117] ~3–4% ATRP/RAFT [124,167,168]
PVC (Polyvinyl Chloride)	- Suspension/emulsion polymerization - Mechanical/chemical recycling - Green plasticizers	- Suspension: 40–70 °C, 0.5–1.5 MPa [138,139] - Emulsion: 40–60 °C [138] - Recycling: 200–300 °C [129,130] - Plasticizers: phthalates vs. bio-based [141,142]	~80–85% suspension polymerization [169] ~10–15% emulsion polymerization [129] <5% recycling/green plasticizers [160,161,162,170,171,172,173]

**Table 5:** Comparative analysis of sustainability metrics, performance, and economic drivers for major commodity polymers.

Polymer	Conventional Route (Yield %)	Carbon Footprint (kg CO_2_ eq/kg)	Carbon Footprint—References with System Boundaries Description	Bio-Based Potential	Main Industrial Driver (Brazil/Global)
PET	90–95	2.1–2.5	Cradle-to-grave (production, use, recycling, disposal) [177]. Cradle-to-gate (raw material extraction to polymer production) [178].	Bio-MEG (sugar cane)	Circularity (bottle-to-bottle)
PE	96–99	1.6–2.0	Cradle-to-gate [179]. Cradle-to-gate with biogenic carbon accounting [180].	Bio-ethylene (ethanol)	Flexible packaging/Bio-PE
PP	95–98	1.8–2.2	Cradle-to-gate [181]. Cradle-to-grave (including recycling) Finding: Recycled PP reduces footprint by up to 75–86% [182].	Bio-PP (glycerol)	Lightweighting (Automotive)
PS	92–96	3.0–3.5	Cradle-to-gate with recycling scenarios [183]. Cradle-to-grave. Finding: Chemical recycling reduces emissions by 37–50% [184].	Styrene from biomass	Insulation/advanced Recycling
PVC	94–97	1.9–2.5	Cradle-to-gate [185]. Cradle-to-grave. Finding: Higher values (~7.8 kg CO_2_ eq/kg) when including use-phase and disposal [186].	Bio-VCM	Construction/durability

**Table 6:** Comparative analysis of technologies and processes, TRL scale, and current status and implementation barriers.

Category	Technology/Process	TRL ^1^	Current Status and Implementation Barriers
Conventional Synthesis	Ziegler–Natta/metallocene polymerization (PE, PP, and PVC)	9	Full commercial operation. Mature and globally optimized technology.
Renewable Monomers	Bio-ethylene (ethanol dehydration—e.g., Braskem)	9	Commercialized. Large industrial scale (Brazil); feedstock cost vs. fossil parity remains a challenge.
Renewable Monomers	Bio-propylene (glycerol or bionaphtha cracking)	7–8	Industrial demonstration. Pilot units operational; yield and cost efficiency are primary barriers.
Recycling	Mechanical recycling (PET, PE, and PP)	9	Commercialized. Global standard; limited by purity and cumulative polymer degradation.
Recycling	Chemical recycling (pyrolysis/thermal depolymerization)	7–8	Scaling-up. Large-scale facilities under construction; high energy intensity and sorting requirements.
Recycling	Enzymatic recycling (PET specific)	6–7	Demonstration. Pilot units (e.g., Carbios); high selectivity but high enzyme costs.
Advanced Catalysis	Late-transition-metal catalysts	4–6	Research/pilot. High functional group tolerance: challenges include thermal instability and catalyst life.
Carbon Capture	CO_2_-based polymers (carbon-to-plastics)	5–6	Prototype/pilot. Emerging technologies; dependent on affordable green energy and carbon capture efficiency.

^1^ TRL Scale: 1–3 (basic research/concept); 4–6 (laboratory/pilot validation); 7–8 (real-scale demonstration); 9 (commercial operation).

## Data Availability

No new data were created or analyzed in this study. Data sharing is not applicable to this article.

## Supplementary Materials

The following supporting information can be downloaded at: https://www.mdpi.com/article/10.3390/polym18081007/s1: Table S1: Descriptions of the different technologies that are used to produce PET around the world; Figure S1: Alternative PET synthesis routes (with no specification regarding the use of catalysts or operational conditions). Terephthalic acid originates from p-xylene produced through bio-based intermediates, such as isobutanol, propane, furane derivatives, benzene, and terpenes. At the same time, ethylene glycol can be obtained from glucose, glycolaldehyde, ethanol, and ethylene obtained from biomass and CO2; Figure S2: SABIC conversion pathways for TPA from HMF, limonene, or muconic acid; Table S2: Comparison of polyethylene polymerization platforms: Ziegler–Natta, Phillips, and single-site catalysts; Figure S3: Polypropylene (PP) overview: properties, global market, main producing companies, applications, and Brazil’s main numbers. References [194,195,196,197,198,199,200,201,202,203,204,205,206,207,208,209,210,211,212,213,214,215,216] are cited in the supplementary materials.
