# Supplementary material for: Safety and Innovation in Conventional Plastics: A Review of Polymer Synthesis and Emerging Technologies
Source: Polymers (Basel). 2026 Apr 21;18(8):1007. doi: 10.3390/polym18081007 (PMC13119562; doi:10.3390/polym18081007)
Supplement: Supplementary file 1 [file polymers-18-01007-s001.zip › polymers-4218076-supplementary.pdf]

Safety and Innovation in Conventional Plastics: A Review of Polymer Synthesis and Emerging Technologies

Derval dos Santos Rosa[1], Helio Wiebeck[2], Alana Gabrieli de Souza[1*], Sueli Aparecida de Oliveira[1], Manoel Lisboa da Silva Neto[2]

1 Center for Engineering, Modeling, and Applied Social Sciences (CECS), Federal University of ABC (UFABC), Santo Andre, Brazil.
2 Metallurgical and Materials Engineering Department (PMT), Polytechnic School of the University of Sao Paulo (USP), Sao Paulo, Brazil
* *alana.gabrieli@ufabc.edu.br / alana_gs@live.com

Supplementary Material

PET and its common synthesis routes

This polymer exhibits excellent thermal stability (-70 - 150 °C), impact strength (19 - 46 MPa), elastic modulus (55 - 100 MPa), tensile strength (40 - 60 MPa), and favorable barrier properties against gases and moisture. These characteristics ensure the preservation of aroma, flavor, and nutritional quality of food products, while also preventing external contamination. Brazil produced approximately 801,000 tonnes of PET virgin resin in 2024, and the leading producers include Alpek Poliéster (450,000 tons/year production capacity) [203], and Indorama Ventures (~550,000 tons/year capacity) [204]. Indorama also expanded its PET recycling capacity in Minas Gerais, Brazil, from 9,000 tons/year to 25,000 tons/year of post-consumer recycled PET resin, aligning with its ambition to build a sustainable global company [205]. Another Brazilian company is Engepack, 100% nacional, operating with the largest and most modern preform PET factories in Brazil [206]. These numbers consolidate the country as the largest PET producer in South America. Additionally, a record recycling level was reported: in 2024, ~410,000 tonnes of PET bottles were recycled, 14% more than 2022. The installed recycling capacity reached ~510,000 tonnes/year, generating revenues of R$ 5.66 billion. Importantly, around 40% of this revenue is distributed along the reverse logistics chain, benefiting cooperatives, waste pickers, and scrap traders.

The esterification is the most common continuous synthesis route, also named direct esterification + liquid phase polycondensation (LCP), requiring stirred reactors and polycondensation columns. For this route, purified (dimethyl)terephthalic acid (TPA) and ethylene glycol (EG) are used. TPA is prepared from p-xylene oxidized in the liquid phase with molecular oxygen, using acetic acid as a solvent and a catalyst system containing cobalt, bromide, and manganese ions [207]. EG is obtained from ethylene, also by catalytic oxidation with ethylene oxide and subsequent hydrolysis. EG and TPA react at 240-260 °C and moderate pressure, generating monoethylene ester (MEG) and bis(2-hydroxyethyl) terephthalate (BHET), and releasing water as a subproduct. Then, polycondensation occurs by depolymerization/dihydroxylation of BHET at 270-280 °C under vacuum, eliminating ethylene glycol as a byproduct to form PET medium- or high-molecular-weight amorphous chains. The catalyst, usually ethylene glycol antimonate or antimony oxide, and process conditions can significantly influence the polymer's properties and efficiency [17]. However, due to the toxicity

of these compounds, there is a tendency to use alternative catalysts from titanium, aluminum, or germanium [22]. Table S1 shows a description of the main technologies used to produce PET.

Table S1. Description of the different technologies that are used to produce PET around the world.

Route	Starting Materials	Key Steps	By-products	Notes
**PTA Route (Direct Esterification)**	Purified terephthalic acid (PTA) + monoethylene glycol (MEG)	1. Esterification at 240-260 °C under pressure - BHET oligomers<br>2. Melt polycondensation at 270-280 °C under vacuum/inert gas - PET melt	Water (H_2O) from esterification, excess MEG from polycondensation	Most common today, high-purity PTA gives high-quality PET; continuous process
**DMT Route (Transesterification)**	Dimethyl terephthalate (DMT) + MEG	1. Transesterification at 150–200 °C with catalysts (e.g., Mn, Co, Zn) - BHET oligomers<br>2. Melt polycondensation as above	Methanol (MeOH) from transesterification, excess MEG	Older route; easier transport/storage of DMT; declining use due to cost/environmental factors
**Integrated PTA–PET**	PX (paraxylene) - PTA (in-plant production) + MEG	1. PTA production and 2. PET polymerization in one complex	Same as PTA route	Reduces logistics cost and contamination risk

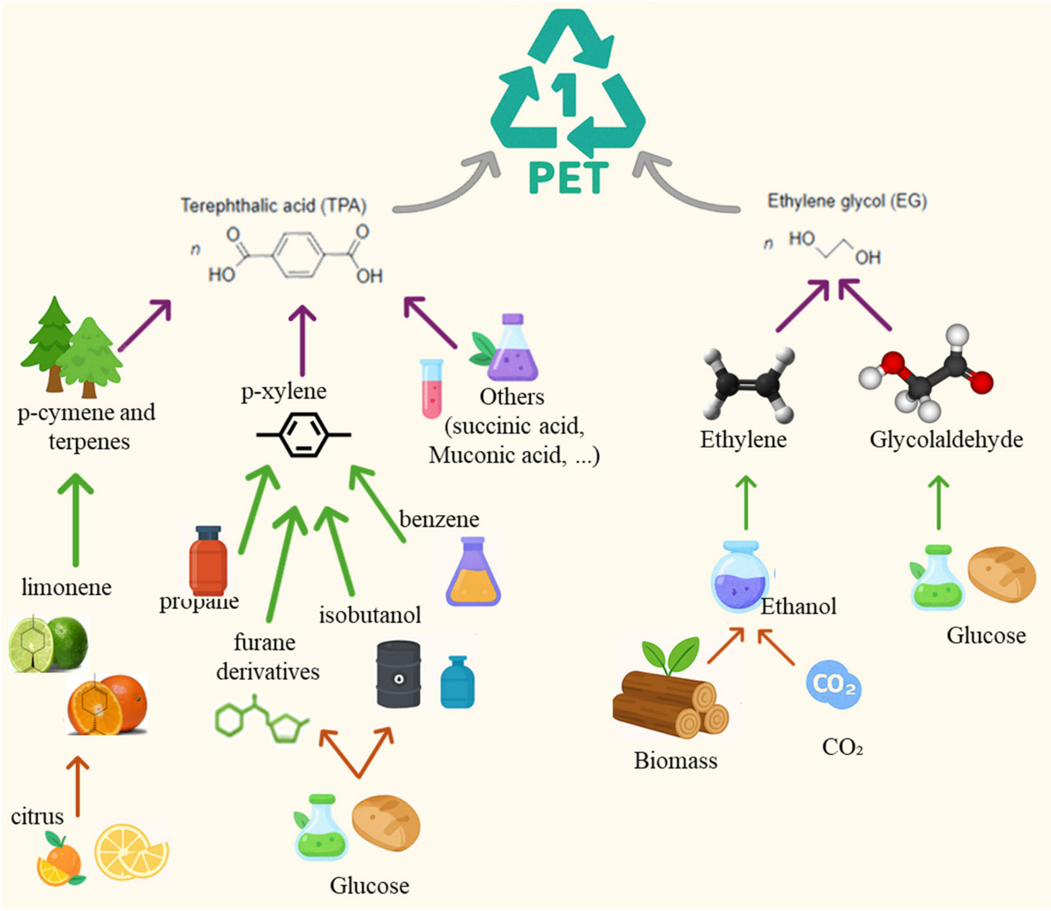

Figure S1. Alternative PET synthesis routes (with no specification regarding the use of catalysts or operational conditions). Terephthalic acid originates from p-xylene produced through bio-based intermediates such as isobutanol,

propane, furane derivatives, benzene, and terpenes. At the same time, ethylene glycol can be obtained from glucose, glycolaldehyde, ethanol, and ethylene obtained from biomass and CO_2.

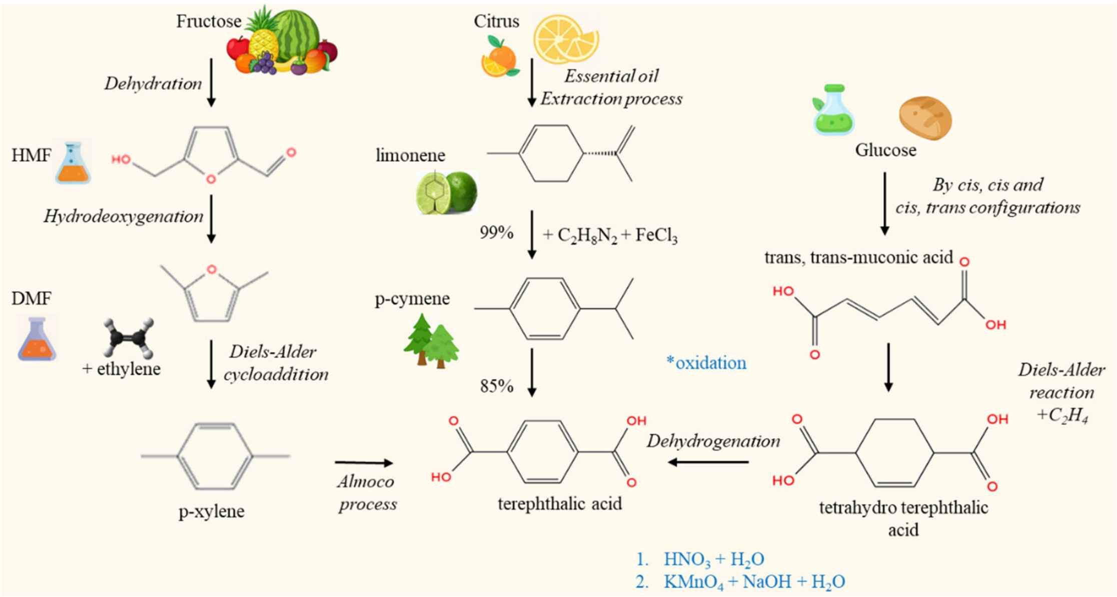

Figure S2. SABIC conversion pathways for TPA from HMF, limonene, or muconic acid.

PE and its common synthesis routes

PE production in Brazil is strongly consolidated in a few large players, with Braskem operating the five major production sites in Brazil, with an annual production capacity exceeding 3.5 million tons/year. This company is also a global pioneer in bio-based PE ("I'm green™" bio-PE), produced from sugarcane ethanol, with a dedicated capacity of ~260,000 tons/year [207]. Other multinational chemical producers can also be cited, such as Dow and Unipar. Considering the PE recycling in Brazil, structural and technological challenges are distinct from those of PET, particularly due to the heterogeneity and predominance as thin-film packaging [208]. National data indicate that the recycling industry as a whole processed nearly 939,000 tons of post-consumer plastics in 2024 [209]. Figure 1b summarizes the main information described in this section.

The Ziegler-Natta (ZN) synthesis was developed in 1953 and industrially scaled in 1955, being considered the most common method. The ZN method occurs under medium pressure (15-30 atm) using organic compound catalytic conditions, resulting in linear PE [210]. Phillips' process was also developed in this 1950s, proposing chromium oxide - silica gel as a catalyst, and several companies acquired licenses, such as Celanese Corporation of America, W. R. Grace & Company, Union Carbide and Carbon Corporation, and M. W. Kellogg Company. Table S2 describes the PE synthesis routes with the main characteristics of each one, and their processing characteristics.

Table S2. Comparison of polyethylene polymerization platforms: Ziegler-Natta, Phillips, and Single-site catalysts.

	**Ziegler-Natta (ZN)**	**Phillips**	**Single-site catalysts**
**Common metals**	Ti (major), V (minor)	Cr on silica; Mo traces in some variants	Zr, Ti (metallocenes); Hf, Fe, Ni (post-metallocenes)
**Support**	$MgCl_2$ or SiO_2	SiO_2 (dehydroxylated/ activated)	Typically, unsupported (homogeneous with MAO/MMAO/boranes) or supported on modified SiO_2

**Cocatalyst / Activator**	Al-alkyls (TEAL, TIBA) + internal/external donors	None for 1st gen Phillips (thermal activation)	MAO, MMAO, $B(C_6F_5)_3$, fluorinated aluminates
**Typical reactor modes**	Slurry (loop), gas-phase (fluidized bed), Solution	Gas-phase, Slurry	Solution (common), slurry/gas-phase when supported
**Primary PE grades**	LLDPE, VLDPE, HDPE; bimodal via dual-reactor or blends	HDPE (high Mw), some LLDPE	LLDPE/VLDPE high-performance; selected HDPE
**Polydispersity**	~3 - 7 (can be multimodal)	~8 - 25 (broad)	~2 - 3 (narrow)
**Comonomer incorporation**	Moderate (1-butene/1-hexene/1-octene)	Low-moderate, less uniform	High and uniform (especially with metallocenes)
**Hydrogen response**	Strong (broad Mv control)	Moderate	Variable to strong
**Density range ($g \cdot cm^{-3}$)**	0.915 - 0.970	0.945 - 0.970	0.880 - 0.945 (VLDPE/LLDPE) and 0.940 - 0.965 (HDPE)
**Branching / Crystallinity**	Linear to branched; control via comonomer and donors	Mostly linear; high crystallinity	Short-chain branching; LCB possible with tailored catalysts
**Typical applications**	Blow molding, films (LLDPE), pressure pipes (HDPE bimodal)	HDPE for blow molding, containers/IBCs, robust blown films	Premium films, sealant layers; specialty HDPE
**Advantages**	Large portfolio, cost-effective, easy bimodal MWD	High impurity tolerance, excellent rheology at high Mw, low catalyst cost	Tailored microstructure, highly reproducible properties
**Limitations**	Medium sensitive to impurities (needs purified monomer/H_2); fine microstructure control depends on donor system	Lower comonomer uniformity distribution	Higher activator cost; sensitivity to impurities; narrow polydispersivity may reduce melt strength
**Representative systems**	$TiCl_4/MgCl_2$ + TEAL + donors	CrO_3/SiO_2 (Phillips), variants with Ti/fluorides	Cp_2ZrCl_2/MAO, CGC, FI-catalysts, α-diimine (Ni/Fe)
**Processing characteristics**			
**Pressure/Temperature**	10 - 30 bar; 60 - 100 °C (slurry/gas)	10 - 25 bar; 70 - 110 °C	Solution 140 - 250 °C; slurry/gas when supported
**MWD multimodality**	High (dual reactor/blends)	Medium (broad MWD; blends for multimodal)	Low - medium (narrow polydispersity; multimodal blends)
**MI/MFR window ($g.10\ min^{-1}$)**	~0.05 - 50	~0.04 - 30	~0.2 - 100 (precise control)
**Sustainability/ Integration**	Compatible with bio-ethylene; accepts hydrotreated pyrolysis oils	Well-suited to reintroduce pyrolysis oils (after hydrotreating); bio-ethylene compatible	Excellent with bio-ethylene; high purity for food-contact; cracker feeds after treatment

Briefly, PE is synthesized from ethylene ($CH_2=CH_2$) via two distinct routes: free-radical polymerization at very high pressure for LDPE, and coordination catalysis at moderate pressure for LLDPE and HDPE. The historical and still highly relevant route is the high-pressure (1,000 to 3,000 bar), high-temperature (180-320 °C) free-radical process conducted in tubular or autoclave reactors with organic peroxides as initiators [211,212]. The free-radical route is characterized by several steps, initiation, propagation, termination, and chain transfer, and the PE chain growth proceeds through radical addition to the ethylene double bond, while chain transfer and backbiting reactions generate both short- and long-chain branching [213]. The reaction results in a polymer with broad molecular-weight distribution and highly branched microstructure, i.e., LDPE with relatively low crystallinity, good transparency, and easy processability [214]. Reactor design, initiator strategy, and residence-time control are the principal levers for tailoring melting index and branching frequency in this route [63].

The second industrial technology involves transition-metal catalyst to coordinate and insert ethylene into a metal-carbon bond, enabling precise control over chain transfer, comonomer incorporation, and molecular-weight distribution. ZN catalysts are a combination of

organometallic cocatalyst with transition metal compounds directly in the polymerization reactor [211,215,216]. Historically, titanium chlorides were favored for their availability and cost, and early (1960s) formulations were obtained by reducing $TiCl_4$ with aluminum alkyls (often in the presence of hydrogel/aluminum), using diethylaluminum chloride as cocatalyst. Nowadays, modern ZN catalysts are high-surface-area $MgCl_2$-supported $TiCl_4$ systems activated by trialkylaluminum (e.g., TEAL/TIBA) and tailored with internal/external electron donors to tune site structure, comonomer response, and hydrogen sensitivity. In this technology, an alkylaluminum cocatalyst first alkylates and partially reduces the precatalyst, generating an alkyl-titanium active site (Ti-R′) with a vacant coordination position. This "active catalyst" is accompanied by the formation of M-Cl. Then, ethylene coordinates to the vacant site via π-interaction. The coordinated olefin undergoes 1,2-migratory insertion into the metal-carbon bond, converting Ti-R′ into Ti-CH_2-CH(R)-, elongating the chain by one monomer unit. After insertion, the metal center re-organizes to recreate a vacant site. Repetition of this cycle produces the polymer chain segment $[–CH_2–CH(R)–]_n$ bound to titanium.

PP and its common synthesis routes

In Brazil, the PP industry is highly consolidated, with Braskem as the dominant producer and the largest polyolefins company in Latin America, operating in multiple units integrated with crackers and downstream converters, supplying homopolymer, random, and impact copolymers. National installed PP capacity totals a few million tons per year, positioning Brazil as a major regional producer [91,207]. According to Abiquim, PP imports reached 339,016 t in 1H 2025; combined Braskem indicators and Abiquim data indicate apparent PP consumption of 933,753 t in 1H 2025. Structurally, PP is a poly(α-olefin) derived from propylene and is obtained as isotactic (iPP) grades via stereospecific coordination catalysis (ZN or single-site), with syndiotactic (sPP) and atactic (aPP) forms accessible under specific catalytic regimes [217,91]. PP combines low density (~0.90 $g.cm^{-3}$), high chemical resistance, good fatigue performance, low moisture uptake, tensile strength ~25 - 40 MPa and elastic modulus ~1 - 2 GPa depending on grade and nucleation/crystallization control [96,218].

According to the literature, the ZN PP polymerization is divided according to the catalyst's generations, since the essential elements for a successful reaction were high catalysts activity and stereospecificity. The zero generation was a combination of aluminum alkyl and titanium and resulted in a polymer with < 50% isotactic content due to the low activity, ~20 g PP/g catalyst. The 1st generation was in-situ preparation of $TiCl_4$ and aluminum metal, resulting in 200 g PP/ g catalyst with isotactic index of ~96%, while the 2nd was important in the production of spherical PP particles, enhancing polymer flowability, and, consequently, applications. The activity increased 5 times, and the stereospecificity in 2-3%. The 3rd generation appeared by the effective combination of $MgCl_2$ and titanium tetrachloride, resulting in double catalyst activity versus 2nd gen, maintaining the same isotactivity, achieving the goals of catalyst residues elimination and atacticity [219]. In the early 1980s, bifunctional phthalate internal donors and silane external donors added precise site-population control, enabling isotacticity ~98% and higher activity (~50 kg PP g^{-1} cat depending on whether productivity is normalized to Ti or total catalyst). These improvements, however, intensified heat release during polymerization. The 4th generation $MgCl_2/TiCl_4$ systems with engineered phthalate/diether donors plus silane external donors set the stereocontrol baseline and H_2 sensitivity, while 5th generation catalysts adopted non-phthalate internal donors (e.g., succinate diesters such as dibutylsuccinate or rac-diethyl-2,3-diisopropylsuccinate) to meet odor goals and tailor MWD [97]. Today's $MgCl_2$-supported $TiCl_4$

and donor packages from 4th and 5th gen constitute the workhorse for iPP homopolymer, random copolymer, and impact PP.

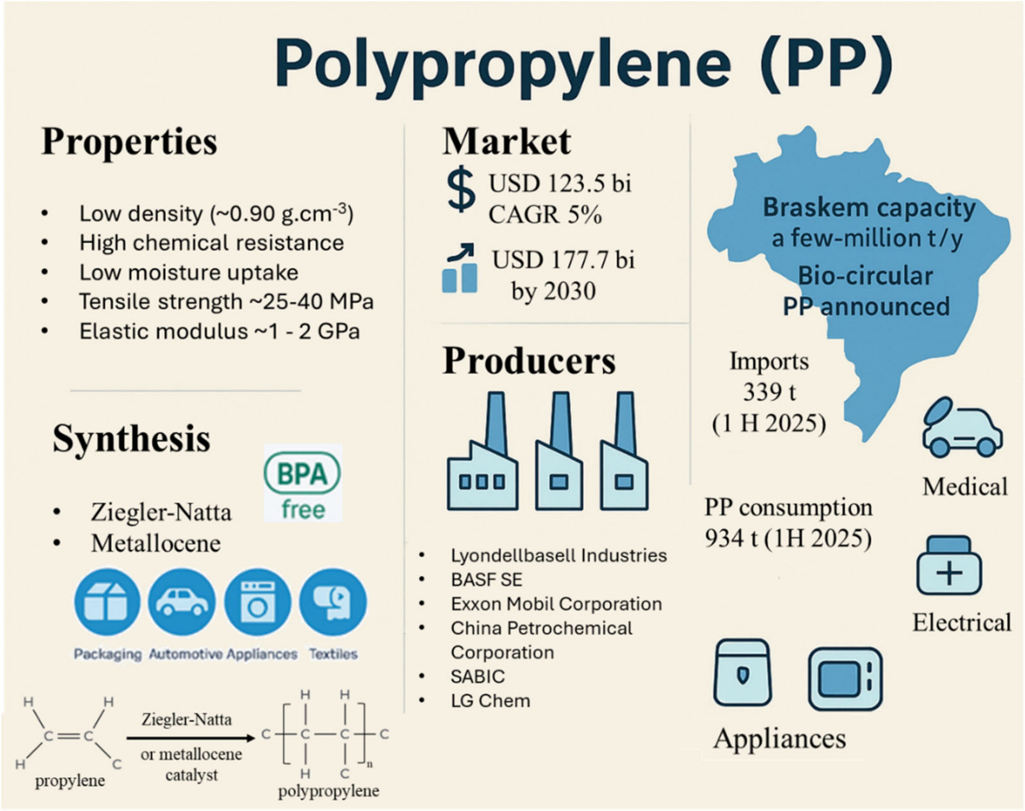

Figure S3. Polypropylene (PP) overview: properties, global market, main producing companies, applications, and Brazil's main numbers.

PS and its common synthesis routes

PS has several structures and its properties are variable according to the application. For rigid food-contact items and disposable ware, PS has optical clarity, stiffness, ease of injection or thermoforming, low moisture uptake, and good electrical insulation. In this case, the properties are: Tg ~ 95-105 °C, elastic modulus ~ 2.3-3.2 GPa, tensile strength ~35-55 MPa, and notched Izod impact strength ~ 100-300 $J \cdot m^{-1}$ [132,220]. In Brazil, Innova S.A. leads PS, operating integrated styrenics assets at Triunfo (RS) and Manaus (AM). Recent disclosures highlight styrene monomer capacity of ~420 $kt.yr^{-1}$ at Triunfo (enabling downstream PS) and a PS capacity debottleneck of ~14 $kt.yr^{-1}$, alongside the launch of ECO-PS® grades with up to 30% post-consumer recycled content. For expanded polystyrene (EPS), the largest PS family segment, Brazil's post-consumer mechanical-recycling rate reached 25.6% (year-base 2023), according to the national monitoring program cited by ABIPLAST/PICPlast, positioning EPS behind PET and HDPE but ahead of several other resins [221].

The predominant PS industrial synthesis method is free-radical polymerization, initiated by heat (Diels-Alder adduct to generate radicals to initiate polymerization) or peroxides. This process follows first-order kinetics at high monomer concentrations, and termination occurs via disproportionation or radical recombination. The final product (GPPS) is inherently brittle, transparent, and amorphous, and can be modified with rubbery monomer or pentane to generate high-impact PS or expandable PS, respectively. Other methods are widely investigated to tailor molecular architecture, including anionic polymerization (precise molecular weight and tacticity),

cationic polymerization (less common due to poor control and side reactions), coordination polymerization (metallocene catalyst to achieve unique electrical and barrier properties), and controlled radical techniques [222].

Controlled radical polymerization was developed to overcome the limitations of conventional radical polymerization, such as high reactivity of chain propagation radicals [223]. These methods include different synthetic methods, such as atom transfer radical polymerization (ATRP), cobalt mediated radical polymerization (CMRP), reversible addition-fragmentation chain transfer (RAFT), iodine transfer polymerization (ITP), stable free radical polymerization (SFRP), and organometallic radical polymerization (OMRP). Despite enabling synthesis of block copolymers, grafted polymers, and complex architectures, it is still limited in industrial application due to cost and complexity [125]. Charge-transfer polymerization (CTP) is a technique used for copolymers and materials with electronic applications, with high cost and restricted to advanced research. From a process view, suspension and emulsion polymerization, applied mainly for EPS production, generates spherical particles that can be expanded later. The polymer obtained shows excellent heat transfer, which is crucial in large-scale processes [169].

PVC and its common synthesis routes

According to the most recent assessment by the Brazilian PVC Institute (IBPVC, 2025), the national PVC market underwent a rebalancing in 2024 marked by declining domestic production and a simultaneous rise in apparent consumption. Brazil maintains an installed PVC capacity of approximately 1.009 million tons per year, underscoring the strategic relevance of its industrial infrastructure [224]. Braskem and Unipar are the leader companies, and the production is led by Braskem, with an installed capacity of approximately 710 - 750 kt/year, distributed across its industrial units in Bahia, Alagoas, and São Paulo. The consumption profile is dominated by the construction sector (~62% of national PVC demand), reflecting the material's strong presence in pipes, profiles, and infrastructure applications. Braskem supplies PVC-S (suspension grade) to multiple sectors. Other important actors in the national PVC value chain include Tigre, Amanco Wavin, Krona, and Fortlev, which convert PVC into high-volume construction and infrastructure products. Brazil also displays a significant recycled PVC economy, with recycling rates in 2024 estimated at ~24% of post-consumer PVC [225]. The growing adoption of PVC recycling technologies, especially mechanical recycling, micronization, and separation of plasticized/non-plasticized PVC, supports circularity targets in the domestic polymer chain. Furthermore, Brazilian converters increasingly incorporate post-industrial PVC recycled (PIR) into non-critical applications, reducing cost and environmental impact.

PVC production relies on chloride monomer (VCM), a colorless gas under normal temperature and pressure. VCM polymerization occurs primarily through free radical mechanisms, typically initiated by organic peroxides or azo compounds under suspension or emulsion conditions [37]. Currently, ethylene chlorination is commonly performed to produce 1,2-dichloroethane (EDC), which undergoes thermal cracking. EDC dehydrochlorination occurs via pyrolysis in cracking furnaces between 500-550 °C.

PVC polymerization occurs in pressurized stirred-tank reactors (50-100 m^3) under temperatures between 40-70 °C, where monomer droplets are dispersed in water by suspending agents (cellulose ethers, PVA) and polymerized via radical initiation. Heat removal is a major engineering challenge, mainly in the bulk process, due to the strong exothermicity (~71 $kJ.mol^{-1}$), requiring efficient cooling. This factor improves the cost due to the equipment need, such as jacketed reactors, reflux cooling, or external heat exchangers to prevent uncontrolled pressure rise

and VCM boiling [137]. As VCM conversion approaches 80–90%, the slurry is stripped, centrifuged, dried, and sieved to control particle morphology.
